# Dynamical Analysis of a Boolean Network Model of the Oncogene Role of lncRNA ANRIL and lncRNA UFC1 in Non-Small Cell Lung Cancer

**DOI:** 10.3390/biom12030420

**Published:** 2022-03-09

**Authors:** Shantanu Gupta, Ronaldo F. Hashimoto

**Affiliations:** Departamento de Ciência da Computação, Instituto de Matemática e Estatística, Universidade de São Paulo, Rua do Matão 1010, São Paulo 05508-090, SP, Brazil; ronaldo@ime.usp.br

**Keywords:** Long non-coding RNA, ANRIL, UFC1, miRNA-34a, Myc, Boolean model, feedback loops, senescence, apoptosis, NSCLC

## Abstract

Long non-coding RNA (lncRNA) such as ANRIL and UFC1 have been verified as oncogenic genes in non-small cell lung cancer (NSCLC). It is well known that the tumor suppressor microRNA-34a (miR-34a) is downregulated in NSCLC. Furthermore, miR-34a induces senescence and apoptosis in breast, glioma, cervical cancer including NSCLC by targeting Myc. Recent evidence suggests that these two lncRNAs act as a miR-34a sponge in corresponding cancers. However, the biological functions between these two non-coding RNAs (ncRNAs) have not yet been studied in NSCLC. Therefore, we present a Boolean model to analyze the gene regulation between these two ncRNAs in NSCLC. We compared our model to several experimental studies involving gain- or loss-of-function genes in NSCLC cells and achieved an excellent agreement. Additionally, we predict three positive circuits involving miR-34a/E2F1/ANRIL, miR-34a/E2F1/UFC1, and miR-34a/Myc/ANRIL. Our circuit- perturbation analysis shows that these circuits are important for regulating cell-fate decisions such as senescence and apoptosis. Thus, our Boolean network permits an explicit cell-fate mechanism associated with NSCLC. Therefore, our results support that ANRIL and/or UFC1 is an attractive target for drug development in tumor growth and aggressive proliferation of NSCLC, and that a valuable outcome can be achieved through the miRNA-34a/Myc pathway.

## 1. Introduction

Long non-coding RNAs (LncRNAs) are defined as a class of non-coding RNAs (ncRNAs) with more than 200 nucleotide lengths. LncRNAs participate in multiple biological processes such as cell-cycle progression, apoptosis and genome stability [1]. In this context, a study of Nie et al. [2] explored the role of the lncRNA antisense noncoding RNA gene at the INK4 locus (hereafter referred to as ANRIL) in NSCLC progression. The study further demonstrated that the overexpression of ANRIL reduces p21 and KLF2 by inducing EZH2 and modulates proliferation in NSCLC while the knockdown of ANRIL regulates apoptosis and cell-cycle arrest by halting the NSCLC progression. On the other hand, recently, Zang et al. [3] found that the long intergenic noncoding RNA UFC1 (hereafter referred to as UFC1) expression level was increased in the tumor tissues and serum of NSCLC patients [3]. Moreover, the high-level expression of UFC1 is associated with tumor progression. Furthermore, Zang et al. [3] observed that UFC1 targets PTEN through the induction of EZH2 while the knockdown (KO) of UFC1 inhibits cancer proliferation and induces apoptosis and cell cycle arrest. They further provided evidence that levels of cyclin D1 and Bcl-2 have significantly decreased in knockdown UFC1 cells. In this way, the study conducted by Zang et al. [3] suggested that UFC1 is involved in the regulation of cell apoptosis and cell cycle arrest in NSCLC cells.

Furthermore, microRNAs (miRNAs) are small ncRNAs that are commonly involved in many biological processes such as cancer progression [4]. They are well-known master regulators of tumor suppression and play an important role in DNA damage response (DDR) through their ability to induce cell cycle arrest and apoptosis [4,5]. The MicroRNA-34a (miR-34a) is a well-known tumor-suppressor miRNA and is downregulated in several types of cancer, including NSCLC [6]. Increasing evidence suggests that Myc is a direct target of miR-34a in breast, glioma, cervical cancer and acute myeloid leukemia (AML) [7,8,9] including NSCLC [6]. In this context, He et al. [6] confirmed the role of miR-34a in the induction of apoptosis and senescence at the G1/S checkpoint by targeting Myc in NSCLC.

Interestingly, recent evidence suggests that miR-34a is the direct target of ANRIL and/or UFC1 [10,11]. In more detail, Wang and colleagues [10] explored the role of the ANRIL/miR-34a axis in AML. They further show that the overexpression of ANRIL enhanced proliferation, migration, and invasion in AML [10]. Moreover, Wang and colleagues [10] found that there is a negative correlation between ANRIL and miR-34a in response to DNA damage. They further observed that the knockdown of ANRIL enhanced miR-34a expression, and induces apoptosis in AML via knockdown Histone deacetylase 1 (HDAC1) [10]. Indeed, HDAC1 is directly targeted by miR-34a, and targeting HDAC1 by miR-34a can modulate apoptosis and senescence in NSCLC cells (see our previous published study [12]). The other piece of evidence comes from the study of Xi et al. [11], which shows that UFC1 had an endorsed role in cervical cancer progression [11], which could regulate its proliferation in HeLa cells. Moreover, they further demonstrated that miR-34a was downregulated [11]. They found that the forced expression of miR-34a could intercept cancer proliferation and trigger cell-cycle arrest and apoptosis in HeLa cells [11]. However, the coordinate regulation between ANRIL and/or UFC1 and miR-34a axis is still unknown in NSCLC.

The interaction among molecules within a cell can be described as a gene regulatory network (GRNs) [13]. GRNs can be defined by graphs with nodes (representing the expression of a gene, or the presence or activity of a protein or cellular process) and edges (describing the interaction connecting the nodes). Different approaches can model GRNs, such as differential equations models, Cellular Automata (CA), Agent-Based Models, and Boolean networks [13]. In the current work, we used Boolean network modeling as it is well suited to capture the silent dynamical properties of GRNs [14,15]. In this formalism, cell fates are associated with model attractors (fixed points or cyclic attractors) whose identification and reachability properties are particularly relevant. Boolean networks do not require a large amount of detailed input information and can be achieved based on the available experiments, which may be incomplete or less quantitative [14]. Boolean networks only require the identification of the interactions and the characterization of the relationship between nodes and the effect of the input signal [14,15]. In the Boolean network, where the node’s state (i.e., “active”/1 or inactive/0) is described by Boolean values (“Active”/ON for activation and “inactive”/OFF for inactivation). These nodes generate their signal by the edges. The state of a node is specified by a Boolean rule that represents the interaction as a logical description [14,15]. The Boolean networks of regulatory networks were intended to simplify the dynamics of complex biological systems [14]. This process provides a qualitative description that captures the advanced features of network dynamics [15]. For example, closed pathways (similar to feedback loops in the continuous model) between two or more nodes in a system can act as regulatory circuits controlling the dynamics of the network [16,17,18]. The description of a molecular regulatory network in a logical frame by a computational–experimental integration process is recognized as a valuable approach to study cell-fate decisions and many biological processes [19,20,21,22]. For more details about Boolean modeling, refer to the Methods.

**Figure 1:**
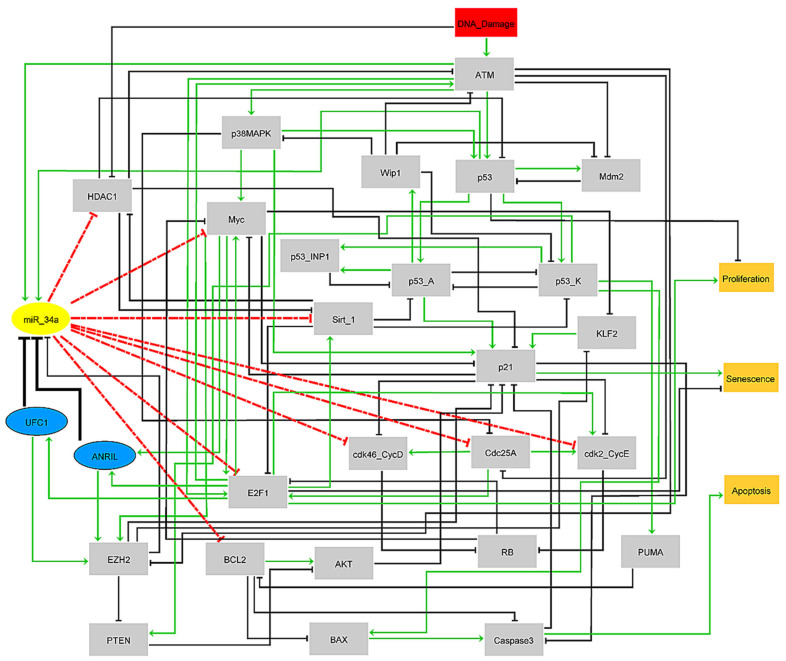
The gene regulatory network for the G1/S checkpoint in response to DNA damage. Arrows denote activations in green and hammer-head arcs represent inhibitions in black, respectively. Dashed hammer-head arcs in red represent targets of miR-34a. The yellow elliptic node represents miR-34a, whereas the blue elliptic nodes represent lncRNAs: ANRIL and UFC1, respectively. The input rectangular node in red denotes DNA Damage. The model outputs in orange in the rectangular nodes are Proliferation, Senescence, and Apoptosis, respectively.

In our previous work [12], we demonstrated that miR-34a regulates a p21-dependent senescence–apoptosis switch by targeting Myc and HDAC1 in NSCLC. In the present study, we extended our previously published model to understand the advanced aspects between these two ncRNAs, i.e., miR-34a and ANRIL and/or UFC1. The expression of lncRNAs such as ANRIL and UFSC1 accelerates tumor growth and progression in NSCLC. In contrast, the increased expression of miR-34a inhibits tumor growth and progression in NSCLC. Using a systems biology approach, we identified a novel mechanism by which the knockdown of ANRIL and/or UFC1 can modulate the miR-34a/Myc pathway at the G1/S checkpoint in NSCLC. Motivated by the facts mentioned above, we propose a Boolean network of the G1/S-checkpoint for NSCLC. To our knowledge, this is the first study in the literature that considers multiple lncRNA versus miRNA in NSCLC (see Figure 1).

## 2. Materials and Methods

### 2.1. Collection of the Public Databases/Tools and Development of the Gene Regulatory Network in NSCLC

We only used databases such as the PubMed, BioGrid (https://thebiogrid.org/) [23], to construct a gene regulatory network involving ANRIL, UFC1 and miR-34a in NSCLC cells. Our interest was in the effect of these two lncRNAs (ANRIL and UFC1) on miR-34a expression. In addition, we identified genes or proteins that were directly targeted by miR-34a. For that, we used three other bioinformatics tools (specific to miRNAs); miRTargetLink 2.0 (https://ccb-compute.cs.uni-saarland.de/mirtargetlink2) [24] and TargetScanHuman 7.1 (http://www.targetscan.org/vert_71/) [25] and LncRRIsearch (http://rtools.cbrc.jp/LncRRIsearch/) [26]. Furthermore, ANRIL and UFC1 directly affect miR-34a activity, triggering its activation of cellular senescence and/or apoptosis at the G1/S checkpoint, or to proliferation due to its inactivation.

Additionally, GINsim 3.0.0b was used for the simulation of Boolean network and visualization of the results [27], which is a Java-based software and is freely available to researchers (http://ginsim.org/downloads) [27]. GINsim algorithms identify all the attractors for the wild-type case, as well as for numerous mutant conditions. In the case of non-deterministic behavior, GINsim assists in the estimation of the probabilities of reaching specific attractors. In this study, we used the Monte Carlo algorithm with ‘exact exit probabilities [27]. The GINsim model file can be found in the Data File S1.

### 2.2. Transformation of the PubMed Literature into a Boolean Network, Rules and Simulations

The Boolean method is based on the performance of a regulatory graph, whereby an individual node describes a molecule, and an individual-directed edge (or arc) implies an activation or repression between two nodes. Nodes are Boolean variables that hold only values 0/Inactive or OFF and 1/Active or ON. Based on the classification of biochemical information, each node in the network is particularized by a logical rule, which defines its activation level in relation to the position of its regulators.

A Boolean model of these ncRNAs, i.e., ANRIL, UFC1, and miR-34a was built by translating the biological interactions described in the gene regulatory network (Figure 1) into Boolean rules (Table S1, PubMed links included). The traditional Boolean operators were used to address these rules “AND”, “OR” and “NOT”.

The attractors are the main outcome of simulations using Boolean networks. The dynamic enforcement of a Boolean model can be characterized by a state transition graph (STG). In this graph, each node specifies the state of the network variables and the arc depicts the transition within these states. STG assists all possible trajectories that can begin from initial state to final state. Terminal nodes that have no outgoing edges are called steady states (or fixed points), while in STGs a set of captured transitions between a fixed set of states defines a cyclic attractor. For updating states, asynchronous updates were considered, which have the potential to describe the non-deterministic performance observed in molecular systems. Furthermore, the dynamics of a gene regulatory network are determined by negative and positive circuits (otherwise known as feedback loops) [17]. Negative circuits can stimulate oscillations while positive ones are responsible for multi-stable dynamics [17]. To test their impact on the dynamics, the Boolean approach facilitates the in silico perturbation of nodes, i.e., constraining them to stay in a fixed position consistent with loss of function (LOF) or gain of function (GOF) experiments, to examine the effect of particular nodes on network dynamics and the resulting phenotype [28].

### 2.3. The Expected Role of These ncRNA in the Boolean Model

Simulations using these two classes of ncRNAs, i.e., lncRNAs (ANRIL or UFC1) and miRNA (miR-34a) in Boolean models should define the biology of NSCLC cells (with or without DNA damage). Indeed, NSCLC cells can be interpreted as having remained in a proliferative state due to the downregulation of miR-34a, i.e., upregulation of ANRIL and/or UFAC1 (in the absence of DNA damage or treatment), whereas in the case when DNA damage is present in the cell, the upregulation of miR-34a is expected, which may suppress proliferation by induction of senescence and/or apoptosis at the G1/S checkpoint as previously reported by He et al. [6]. As a result, three fixed points/steady states (attractors) are expected.

### 2.4. Molecular Mechanisms Mediating ncRNAs at the G1/S Checkpoint in NSCLC

Cell-fate determinations such as senescence, autophagy, and apoptosis are stimulated by DNA damage at both G1/S and G2/M cell cycle checkpoints [16,29,30]. DNA double-strand breaks (DSBs) can be caused by the radiomimetic chemicals or reactive oxygen species (ROS) or radiation, interestingly, recent evidence suggested [31] that knockdown of EZH2 expression (which has a central role in the studies of Nie et al. [2] and Zang et al. [3]) robustly and rapidly induces 53BP1 DNA damage foci [32], leading to DNA damage in cells [31], which triggers autophosphorylation of ATM (ATM serine/threonine kinase) at serine 1981 by initiating its kinase activity [33]. Downstream phosphorylation on the ATM pathway drives the activation of p53 in response to DNA damage. ATM directly induces Mdm2 phosphorylation, thereby reducing the activity of Mdm2 and resulting in the strong induction of p53 [34]. In our model, p53 was characterized by different nodes, depending on its specific phosphorylation states. The p53 node is associated with its interaction with Mdm2 [34], which is directed to activate p53-A and p53-K [35]. p53-A defines p53 phosphorylated at Ser-15 and Ser-20, which activates p21 and Wip1. Conversely, p53-K describes p53 additional phosphorylation at Ser-46, through BCL2-associated X, the apoptosis regulator (Bax) leads to the activation of the apoptotic pathway [35,36]. Both p53-A and p53-K are linked by a positive circuit, and the conversion among them p53-A and p53-K are regulated by Wip1 and the tumor protein p53 inducible nuclear protein 1 (p53-INP1) [35]. ATM [37] and p53 [38] can induce miR-34a expression in response to DNA damage in NSCLC.

**Figure 2:**
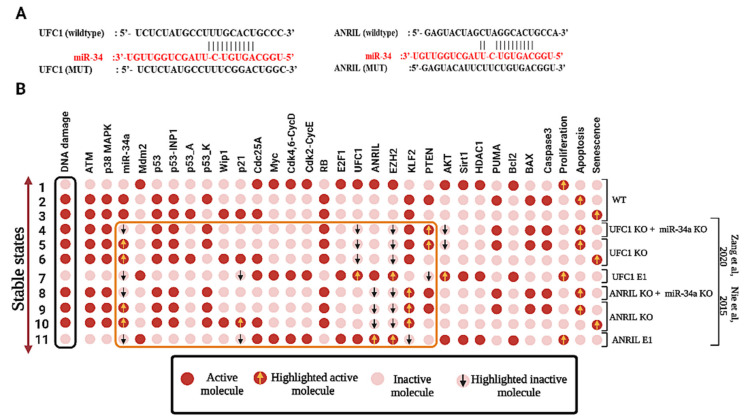
Binding sites of miR-34a and wild-type case attractor of the Boolean networks. (**A**) On the right, predicted binding sites of miR-34a within ANRIL, whereas on the left within UFC1 (**B**) The stable states or fixed points identified for different scenarios are: WT, UFC1 KO + miR-34a KO, UFC1 KO, UFC1 E1, ANRIL KO + miR-34a KO, ANRIL KO, ANRIL E1, miR-34a KO, miR-34a E1 and Myc KO. Gain-of-function (GoF) and loss-of-function (LoF) perturbations correspond to its referential experiments [2,3]. The leftmost column shows the DNA damage level (highlighted in the black box) and the rightmost column presents the model outputs: proliferation, senescence, and apoptosis. Each line represents a fixed point corresponding to the input. Each cell/dot or circle represents a molecule. Light red cells indicate a zero value, whereas red ones indicate activation (value 1). Molecules that are involved in the regulation of miR-34a according to its referential studies (highlighted in the orange box). Orange arrows indicate up-regulation of the molecule, whereas the inverted black arrow represents the downregulation of the molecule, respectively.

In the G1/S checkpoint, miR-34a is required for the induction of the G1/S checkpoint (see our previous published study [12]). Activated miR-34a directly target Myc, HDAC1, Sirt-1, E2F1, Cdc25A, BCL2, CDK4/6-cyclin D1 complex (cdk46-CycD) and CDK2/cyclin E2 (cdk2-CycE) [39]. However, its expression is downregulated by lncRNAs such as ANRIL [2] and UFC1 [3], (see Figure 2A). In this connection, UFC1 expression was initiated by E2F1 [11], whereas ANRIL expression was regulated by both E2F1 [40] and Myc [41]. Interestingly, both E2F1 [42] and Myc [6] are well-known targets of miR-34a. Therefore, closed pathways exist between these molecules such as miR-34a/E2F1/ANRIL, miR-34a/E2F1/UFC1, and miR-34a/Myc/ANRIL. Additionally, EZH2 also directly inhibits miR-34a expression [43]. Furthermore, EZH2 plays a central role in both studies [2,3], i.e., UFC1 promotes the activation of EZH2 to target PTEN [3] (PTEN is a well-known inhibitor of the AKT pathway [44]) while ANRIL facilitates activation of EZH2 to target KLF2 [2] (KLF2 is an upstream regulator of p21 [45]). Thus, using the biochemical information mentioned above, we propose a Boolean model for the G1/S checkpoint in NSCLC cells considering the effect of miR-34a by lncRNAs such as ANRIL and UFC1 in NSCLC.

## 3. Results

### 3.1. Boolean Model and Its Wild-Type Case Attractors

The model includes 32 nodes representing proteins and non-coding RNAs (ANRIL/UFC1 and miR-34a) and 94 direct interactions amongst them. The model has a single input, DNA damage (Figure 1). The blue elliptic nodes represent lncRNAs: ANRIL and UFC1 and a yellow elliptic node signifies miR-34a, whereas other proteins are in gray rectangular nodes. The additional rectangular nodes in orange represent model outputs (Proliferation, Senescence, and Apoptosis). Arrows denote activation in green and hammerhead connectors inhibitions are in black, respectively. See Figure 1.

The model presents three fixed points or states for the wild-type (WT) dynamics which are related to a different phenotype, as shown in Figure 2B. The first state represents a proliferative state (corresponding to the input: DNA Damage = OFF), i.e., no cycle arrest is observed, only the following cell-cycle promoters are activated: CDK46/CycD, CDK2/CycE, Cdc25A, Myc, ANRIL, and UFC1. The other two states induced by the same initial input are: DNA Damage = ON, which corresponds to a stochastic behavior known as a bistable state involving two p53-responsive cellular phenotypes and Senescence and Apoptosis, whose states are described by p53-A and p53-K, respectively. For more details see [35] and our previously published studies [12,16,29,30,46].

### 3.2. miR-34a Is a Downstream Target of ANRIL and/or UFC1 in NSCLC

Recently, Nie et al. [2] explored the role of ANRIL in NSCLC. These authors further demonstrated that the overexpression of ANRIL reduces p21 and KLF2 by inducing EZH2 and modulates proliferation in NSCLC. Besides, Zang et al. [3] investigated the contribution of UFC1 in NSCLC cancer progression. They found that UFC1 targets PTEN through the induction of EZH2 while the knockdown (KO) of UFC1 inhibits cancer proliferation and induces apoptosis and cell-cycle arrest. Although, both studies (Nie et al. [2] and Zang et al. [3]) did not perform miR-34a in their experiments. However, growing evidence suggests that ANRIL and/or UFC1 can regulate miR-34a expression [10,47]. Therefore, we investigated the influence between miR-34a and ANRIL/UFC1 in response to DNA damage, which is our main aim for the current work. To do this we conducted the perturbation of miR-34a and/or along with ANRIL/UFC1 (as can be seen in Figure 2B in the orange box). The knockdown of UFC1 and/or knockdown of ANRIL along with forced knockdown miR-34a abrogates senescence and induces only the apoptotic phenotype, which is partially in agreement with Zang et al. [3] and Nie et al. [2]. Interestingly, in the perturbation’s simulation analysis, we observed that the knockdown of UFC1 and/or ANRIL (single knockdown of each lncRNA or double knockdown of both lncRNA) enhanced miR-34a expression (see in Figure 2B highlighted in an orange box). In addition, we also performed the gain-of-function (GoF) of ANRIL and/ or UFC1 to observe the effect on miR-34a expression. Interestingly, we found that the overexpression (E1) of ANRIL and/or overexpression (E1) of UFC1 diminished miR-34a expression induces proliferation. These results suggested that UFC1 and/or ANRIL may act as miRNA sponges to repress miR-34a expression in NSCLC. Interestingly, our results are in good agreement with experimental studies [10,47].

### 3.3. Calibration between In-Silico Perturbations versus Experimental Studies

To address the phenotype probabilities for the single node perturbation, in a similar method to that performed in an experimental study by Nie et al. [2] and by Zang et al. [3] who used the loss-of-function (LoF) of ANRIL and the LoF of UFC1, respectively. To begin with the method of Nie et al., in which, for the observation of cell viability, they used the H1299 and SPC-A1 cells transfected with si-ANRIL (3000 cells/well) which were grown in 96-well plates and cell viability was assessed every 24. The si-ANRIL or si-NC was transfected into H1299 and SPC-A1 cells were grown on 6-well plates to confluency and transfected using Lipofectamine 2000 (Invitrogen) at 48 h after transfection by trypsinization. For apoptosis, the double staining with FITC–Annexin V and Propidium iodide (PI) were performed using the FITC–Annexin V Apoptosis Detection Kit. For cell cycle analysis, it was stained with PI using the CycleTEST PLUS DNA Reagent Kit. On the other hand, in the experimental study of Zang et al., the A549 cells were seeded into 6-well plates (2  ×  105/well) and cultured in 37  °C incubators overnight. Both the siRNAs and shRNAs were transfected with NSCLC cells by applying LipoFiter transfection reagent. At 6 h after transfection, cells were changed to a complete medium and then cultured for another 30 h. Cell-cycle analysis was conducted at 36 h after transfection by a cell cycle detection kit. The collected cells were fixed in 95% ethanol overnight and then stained with 50 μg/mL PI for 30 min in the dark. Flow cytometry was used to calculate the percentage of the cells in varying phases. For more detail see the study of Nie et al. [2] and Zang et al. [3].

**Figure 3:**
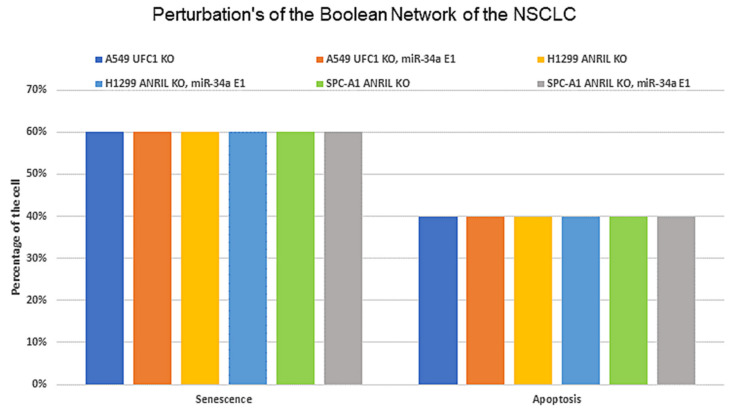
Probabilities of perturbed cases of Boolean networks using Monte Carlo simulations (100,000 runs) and comparison with experimental studies by Zang et al. [3] and Nie et al. [2]. Each bar represents a specific case of the perturbation. As such, the first case is UFC1 KO in A549 cells, the second case is UFC1 KO along with miR-34a E1 in A549 cells. The third case is ANRIL KO, while the fourth case is ANRIL KO together miR-34a E1 in H1299 cells. The fifth case is ANRIL KO in SPC-A1 cells, whereas the sixth is ANRIL KO along with miR-34a E1 in SPC-A1 cells. E1 represents gain-of-function (GoF) and knockdown (KO) represents loss-of-function (LoF), respectively. See Section 3.3 for more information.

Since we aimed to uncover the crucial role of miR-34a through the knockdown (KO) of each of these lncRNAs, we conducted perturbation of the single node using ran Monte Carlo simulations with 100,000 runs. To perform this meant the knockdown (KO) of UFC1 (according to Zang et al. [3]) and running Monte Carlo simulations with 100,000 runs and then, another perturbation for knockdown (KO) of UFC1 was performed, together with the overexpression (E1) of miR-34a after which the Monte Carlo simulations were performed again with 100,000 runs. The results are presented in Figure 3. We repeated the same process of perturbations for the ANRIL., i.e., we used perturbation for the single node knockdown (KO) of ANRIL (according to Nie et al. [2]) and ran Monte Carlo simulations with 100,000 runs and next, another perturbation for knockdown (KO) of ANRIL collectively with the ectopic expression (E1) of miR-34a with Monte Carlo simulations with 100,000 runs. In more detail, according to Zang et al. [3], UFC1 knockdown (KO) produces 60% arrest and 40% apoptosis in DDR. We also found that the perturbation of knockdown (KO) of UFC1 along with overexpression (E1) of miR-34a produces 60% senescence and 40% apoptosis, which is identical to that observed for the A549 cell by Zang et al. [3]. Next, we performed perturbations according to Nie et al. [2] for the knockdown (KO) of ANRIL and ran Monte Carlo simulations with 100,000 runs. Knockdown (KO) of ANRIL induces 60% arrest and 40% apoptosis in H1299 cells and 70% arrest and 30% apoptosis for SPC-A1 cells, respectively. Again, we conducted the perturbation of ANRIL KO together with miR-34a E1 and ran Monte Carlo simulations with 100,000 runs, and we found that overexpression (E1) of miR-34a together with knockdown (KO) of ANRIL induces 60% senescence and 40% apoptosis, which is very similar to the observations of Nie et al. [2] for H1299 cells. Finally, we compared the perturbations for SPC-A1 cells. We found that miR-34a (E1) and ANRIL (KO), could generate almost equal probabilities according to the observations of Nie et al. [2] for SPC-A1 cells. As can be seen in Figure 3, our results are in very close agreement with these experimental studies [2,3]. These results further confirmed that UFC1 and/or ANRIL could regulate miR-34a expression in NSCLC cells. For more detail see Figure 3.

In addition, we calculated, in silico, the probabilities of each phenotype for the wild-type case when the input (DNA Damage) of the model is ON. We used the Monte Carlo algorithm in GINsim with 100,000 runs (see section Methods). For the WT case, we obtained 80% for apoptosis and 20% for senescence.

### 3.4. Activation of miR-34a Inhibits NSCLC Progression through the Targeting of Myc

**Figure 4:**
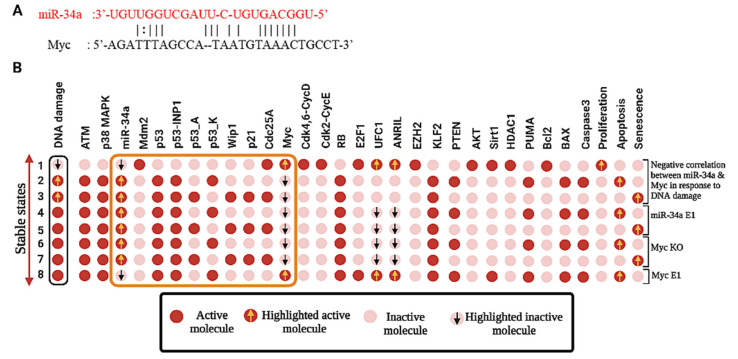
Duplex structure of miR-34a and model perturbations. (**A**) Binding site between miR-34a and Myc. (**B**) Perturbations of miR-34a & Myc in response to DNA damage, miR-34 E1, Myc KO and Myc E1. Gain-of-function (GoF) and loss-of-function (LoF) perturbations corresponding to knockdown (KO) and overexpression (E1) of their referential experiments (He et al. [6]). The leftmost column shows the DNA damage level (highlighted in the black box) and the rightmost column presents the model outputs, including proliferation, senescence, and apoptosis. Each line represents a fixed point corresponding to the input. Each cell/dot or circle represents a molecule. Light red cells indicate a value of zero, whereas red indicates activation (value 1). Activity of miR-34a on the Myc expression as suggested by [6] (highlighted in the orange box). Orange- arrows indicate up-regulation of the molecule, whereas the inverted black arrow represents the downregulation of the molecule, respectively.

To investigate the downstream molecular mechanisms of miR-34a in response to DNA damage in NSCLC as suggested earlier by He et al. [6] using bioinformatics tools, the 3′-UTR (untranslated region) of the Myc gene was predicted to bind with miR-34a (Figure 4A). Interestingly, He et al. [6] show that there is a negative correlation between miR-34a and Myc in the DDR. Furthermore, ectopic expression (E1) of miR-34a suppressed proliferation by knockdown (KO) of Myc in response to DNA damage and induced senescence and apoptosis. Similarly, knockdown (KO) of Myc triggers senescence and apoptosis. Whereas the overexpression (E1) of Myc diminishes miR-34a expression and triggers only apoptotic cell death in DDR. We tested this through perturbation analysis, for which the results are presented in Figure 4B, highlighted in the orange box. As we can see in Figure 4B, the expression of lncRNAs is declined in miR-34a overexpressed or Myc knockdown cells (we have highlighted them in the inverted arrow), whereas Myc overexpressing cells boost the expression of both lncRNAs but diminish miR-34a expression. Our results are in line with those of He et al. [6] and suggest that the activation or overexpression of miR-34a inhibits tumor growth and progression of NSCLC by targeting Myc. However, similar to Nie et al. [2] and Zang et al. [3], He et al. [6] did not consider any lncRNA in their study. Therefore, we used these three experiments [2,3,6] as the main reference for our study (see Table 1). The technique used to build the model was to specifically set up the model to confirm the information provided by each study, for example, one study focused only on the related miRNA, ignoring lncRNAs [6], whereas the other two studies emphasize the role of ANRIL and UFC1 but omit the miRNA [2,3]. Thus, in the present work, we provided a significant advantageous association between these studies. As can be seen in Figure 2B, Figure 3 and Figure 4B, our results are in excellent agreement with those of Nie et al. [2], Zang et al. [3], and He et al. [6]. Taken together, our Boolean Network model has observed the fact that knockdown of ANRIL and/or UFC1 may modulate the biological functions of NSCLC cells by targeting the miR-34a/Myc pathway.

### 3.5. Biological Circuits and Perturbation Analysis

GINsim identified only 28 operative biological circuits, i.e., circuits that actively influence the network dynamics. However, we selected only 16 of those containing a maximum of three elements, as several were already investigated experimentally, see Table 2. (For more information about functional circuits, see Table S2).

Three circuits, out of these 16, are novel circuits, including miR-34a/E2F1/ANRIL, miR-34a/E2F1/UFC1, and miR-34a/Myc/UFC1. Since we wanted to reveal the role of the miR-34a/lncRNA axis in NSCLC, we analyze only those circuits where miR-34a and/or lncRNA were included in this work. Interestingly, the biochemical interactions defining these circuits are well-known in the literature for NSCLC cells (Table 3); although, their functionality in the G1/S checkpoint mechanism is yet experimentally unknown. Then, we decided to investigate, using the perturbations, whether these three circuits were important to regulate the phenotypes. To do this, we used circuit component perturbations to determine which circuits are required to induce phenotype such as, apoptosis and/or senescence, the results are shown in Table 4. As you can see that the perturbation overexpression (E1) of miR-34a alone and/or along with ANRIL/UFC1, induces senescence and apoptosis. On the other hand, the remaining perturbations overexpression (E1) of ANRIL/UFC1 alone and/or together with E2F1 or Myc, triggers only apoptosis phenotype. In this way, these results indicate that these three new biological positive circuits can contribute to blocking cancer progression by regulating senescence and apoptosis at the G1/S checkpoint. For more details see Table 4.

### 3.6. Workflow of the Boolean Network Construction and Validation

We investigated the dynamics of the Boolean Network by comparing the outcomes of the perturbed network to the observed phenotypes of mutants located in experimental data. Our Boolean Network characterizes the regulatory network of the G1/S checkpoint of an NSCLC cell, specified via the biochemical interactions and experiments involving the molecules in Table S1.

**Figure 5:**
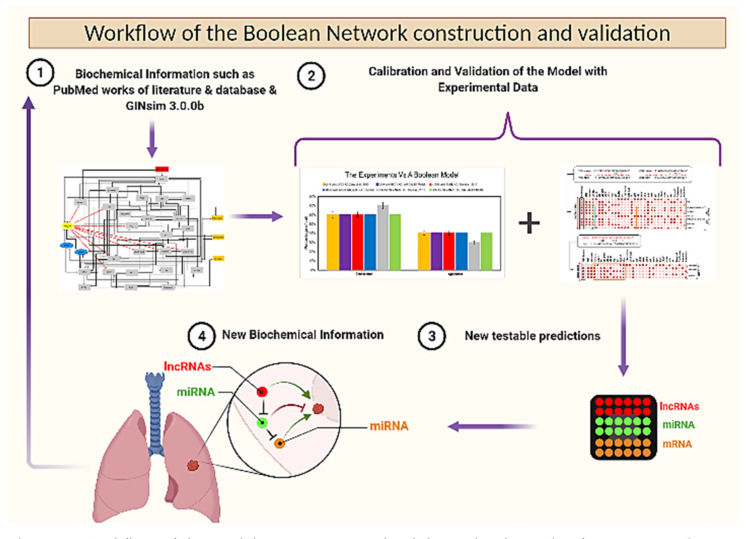
Workflow of the model construction and validation by the cycle of 1 to 4-steps. Step 1, The model was simulated using GINsim 3.0.0b, based on the biochemical information provided by PubMed works of literature and databases. In Step 2, we verified the coherence between the outcome produced by the perturbed nodes and experimental observations using the same perturbations. Step 3, Once the model was collaborated with its referential studies we decided to perform “what if?” scenarios i.e., the hypotheses derived from the “what if” simulations can be used to design-focused experiments. In Step 4, these experimental designs lead to new in-vivo/in-vitro experiments in NSCLC as we see in Step 1. Worth noting is that this analysis was performed using the data used to build the network (see Table S1). To validate the Boolean Network (see Table 1), we used experimental studies that were not utilized in its construction.

The Network was additionally validated in comparison with biochemical data separated from the information in Table S1 used to build the model, and they are shown in Table 1 (see Figure 5). In more detail, as can be seen in Figure 2B, Figure 3, and Figure 4B, once our model collaborated with experimental results and produced the same results that were followed in each experimental study [2,3,6], we decided to conduct “what if?” scenarios i.e., the assumptions derived from the “what if” simulations can be used to design experiments. For instance, according to our Boolean network, a negative correlation between ANRIL and/or UFC1 and miR-34a occurs in response to DNA damage. Second, the knockdown of ANRIL and/or the knockdown of UFC1 triggers the activation miR-34a in DNA damage response. For more details about the predictions of the Boolean Network model, see File S1.

Additionally, the “what if” simulations outcomes can even suggest experiments that would not have been clear before the Network. In more detail, recently, Lu et al. [57] (review study), indicated that targeting single lncRNA analysis is not enough for use as a clinical indicator in NSCLC. Therefore, the combination of two or more lncRNAs for the diagnosis of NSCLC shows a more reliable result and sensitivity from clinical aspects. Hence, we propose a new experimental outline based on model, (1) consisting of miR-34a as an inhibitor of the E2F1/Myc pathway (main inducers of ANRIL/UFC1), which can block cancer progression and modulate apoptosis and senescence. (2) Knockdown (KO) of ANRIL and/or (KO) of UFC1 can trigger functionally stable miR-34a expression.

The possible outcome of this experiment would be a suppression of tumor growth and proliferation in NSCLC. To test this outcome, we suggest these potential strategies predicted by the model:

- UFC1 (KO), miR-34a (E1) → Apoptosis and Senescence;
- ANRIL (KO), miR-34a (E1) → Apoptosis and Senescence;
- ANRIL (KO), UFC1 (KO) → Increased expression of miR-34a.

As such, experimentally testable model predictions are the key outcomes of Boolean Network models, which can lead to new insight into the molecular and biophysical mechanisms underlying the biological process. In turn, the new biological insights lead to new predictions that can be explored and analyzed with Boolean models, resulting in an iterative experimental and modeling research cycle (see in Figure 5).

## 4. Discussion

In this study, we investigated molecular mechanisms involving ANRIL and/or UFC1 and miR-34a at the G1/S checkpoint in NSCLC (see Figure 1). ANRIL and UFC1 are up-regulated and play a crucial role in tumor progression in NSCLC [2,3]. Contrary to this, miR-34a is a well-known tumor suppressor and down-regulated in many types of cancers including NSCLC [6]. Indeed, in the DDR, the overexpression of miR-34a inhibits proliferation by targeting Myc and induces apoptosis and senescence in NSCLC [6]. Two recent studies revealed a new induction mechanism of arrest/apoptosis in NSCLC by knockdown (KO) of UFC1 and by knockdown (KO) of ANRIL [2,3]. However, both studies overlooked the dynamic role of miR-34a in NSCLC [6,12]. To our knowledge, this is the first study that considers the two different lncRNA (ANRIL and UFC1) and one miRNA (miR-34a) and unveils the new regulatory role of miR-34a in NSCLC.

Recently, the studies of Nie et al. [2] and Zang et al. [3] confirmed that ANRIL and UFCA1 can each regulate the proliferation and knockdown of lncRNA (UFC1 and ANRIL) and induce arrest and apoptosis at the G1/S checkpoint in NSCLC. Nevertheless, observations from both studies could not uncover the molecular mechanism linking miR-34a in the induction of cell-cycle arrest and/or apoptosis-like phenotypes. In this context, our results explored a detailed molecular mechanism and explained that the knockdown (KO) of ANRIL and/or knockdown (KO) of UFC1 could regulate miR-34a expression, and once miR-34a expression is triggered in response to DNA damage, it directly inhibits Myc expression. Myc is a well-known inhibitor of p21 activity in NSCLC. Previously, it was proved that p21 acts as a switch between senescence and apoptosis in NSCLC. Thus, activated miR-34a inhibits Myc that can regulate a p21-dependent senescence–apoptosis switch at the G1/S checkpoint in NSCLC. In this way, our study focused on miR-34a and ANRIL/UFC1 effects following the available experimental studies and uncovered the extraordinary mechanisms associated with these molecules in NSCLC.

Lately, many studies have postulated that targeting ANRIL and/or UFC1 can regulate cancer proliferation through the enhanced expression of miR-34a. In this fashion, Dong et al. [56] posed the role of the ANRIL/miR-34a axis in glioblastoma multiforme (GBM). They further demonstrated that ANRIL was significantly up-regulated and miR-34a was downregulated in GBM. Moreover, ANRIL knockdown suppressed cell proliferation and promote apoptosis by GBM by the miR-34a/Sirt1 pathway [56]. Recently, Wang and colleagues [10] explored the role of ANRIL/miR-34a in acute myeloid leukemia (AML). They further demonstrated that overexpression of ANRIL enhanced proliferation, migration, and invasion in AML. Moreover, Wang and colleagues [10] found that there is a negative correlation between ANRIL and miR-34a in DDR. They further observed that the knockdown of ANRIL enhanced miR-34a expression, and induces apoptosis in AML via the knockdown of HDAC1 [10]. Indeed, HDAC1 is directly targeted by miR-34a, and targeting HDAC1 by miR-34a can modulate apoptosis and senescence in NSCLC cells [12].

Recently, the study of Xie et al. [47] revealed that UFC1 had an oncogenic role in breast cancer tissue, which could trigger proliferation, invasion, migration, and EMT. Furthermore, miR-34a was downregulated in the same cells. Xie et al. [47] demonstrated that the knockdown of UFC1 can inhibit proliferation and promote apoptosis of cancer cells by upregulation of miR-34a. On the other hand, Xi et al. [11] showed that UFC1 had a supporting role in cervical cancer progression by regulating proliferation in HeLa cells. Moreover, they further showed that miR-34a was downregulated. They found that the forced expression of miR-34a could inhibit cancer proliferation and trigger cell cycle arrest and apoptosis in HeLa cells [11]. All these studies closely support our results that knockdown of ANRIL and/or UFC1 can markedly inhibit proliferation and induce apoptosis and/or senescence through the miR-34a/Myc axis at the G1/S checkpoint in NSCLC.

Previously, we have shown that miR-34a regulates the p21-dependent senescence–apoptosis switch by targeting Myc and HDAC1 in NSCLC [12]. Indeed, both Myc and HDAC1 are inhibitors of p21 activity [58,59]. Consequently, the overexpression of miR-34a regulates p21 expression by targeting Myc [60] and HDAC1 [48]. Furthermore, increased expression of p21 triggers senescence and inhibits apoptosis [12]. In this way, miR-34a governs the senescence–apoptosis switch in NSCLC in a p21-dependent manner [12]. Understanding the landscape between the lncRNA/miRNA axis in NSCLC and their dynamic behavior in gene regulatory networks is quite complex. To understand this scenario, a systematic understanding of this process is expected. For that, in the present work, we used our previously published Boolean model [12] and extended it according to the available experimental results concerning the lncRNA/miRNA axis at the G1/S checkpoint in NSCLC (see Figure 2B). In case of a lack of DNA damage, the model predicts only a single stable state denoted by a proliferative phenotype that is supported by these experimental studies [2,3,6]. In the presence of DNA damage, bistable dynamics were observed, which coincide with two p53-responsive cellular phenotypes: senescence or apoptosis [6] (see Figure 2B). Undoubtedly, both phenotypes are observed in NSCLC in DDR [6]. The NSCLC cell model was validated according to the experimental results using gain-of-function (GoF) and loss-of-function (LoF) perturbations of its components (see Figure 2B and Figure 4B and Table 1) from various experimental studies [2,3,6]. Additionally, as can be seen in Figure 2B, Figure 3, and Figure 4B, once our Boolean network correlates with the experimental results and returns the same results that were observed per experimental study [2,3,6], we ran the “What if?” scenario i.e., assumptions resulting from “what if” simulations can be served for designing experiments. These simulation results may also suggest experiments that would not have been clear before the network. For example: according to our model, in response to DNA damage, knockdown (KO) of ANRIL and/or knockdown (KO) of UFC1 regulates NSCLC progression via the activation of miR-34a expression in NSCLC. See Table 1 (indicated by a question mark) and File S1 for more details of Boolean network predictions.

**Figure 6:**
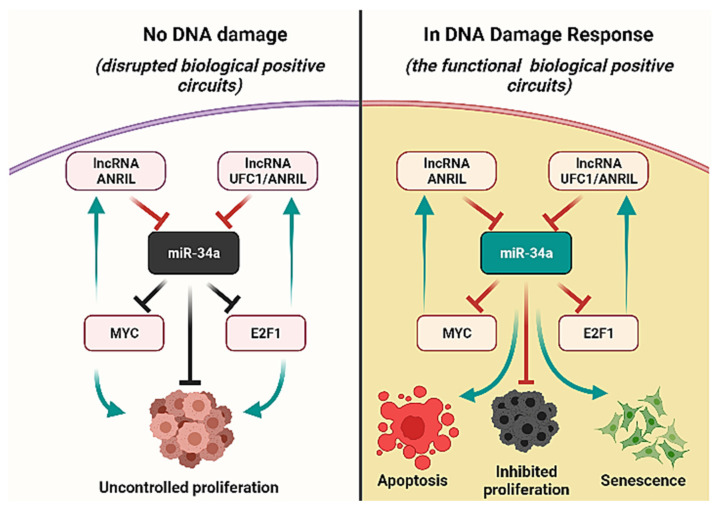
Functional Versus Disrupted positive circuits in NSCLC. The left-hand side: disrupted positive circuits due to the lack of DNA damage in NSCLC cells, i.e., inactivation of miR-34a expression, consequently promoting uncontrolled proliferation. On the Right-hand side: In response to DNA damage, the positive circuits between (miR-34a/Myc/lncRNA ANRIL, miR-34a/E2F1/lncRNA ANRIL, and miR-34a/E2F1/lncRNA UFC1) are stimulated. Activated miR-34a inhibits Myc and E2F1 i.e., inducers of lncRNA ANRIL and lncRNA UFC1. Targeting Myc and E2F1 by the miR-34a prevented tumor growth through the induction of senescence and apoptosis in NSCLC cells. In this way, these positive circuits playing an important role to inhibit proliferation in DNA damage response. Green Arrows represent activation, whereas red hammer-head arrows represent inhibition. Black hammer-head arrows represent non-functional/inactivation of miR-34a, respectively.

Moreover, in GRN, Biological circuits play an essential role. Indeed, GRN is a combination of multiple positive and negative circuits. These circuits can capture and represent the dynamics of the biological system [16]. In such a context, we found three new positive circuits (miR-34a/E2F1/ANRIL, miR-34a/E2F1/UFC1, and miR-34a/Myc/ANRIL). We also provided evidence of these circuits according to their interactions that have exist in NSCLC (for more details see Table 3). Furthermore, we analyzed these circuits (see Table 4) and performed the perturbation of each circuit component. Interestingly our results indicate that these new circuits are important for modulating cancer progression by the induction of senescence and apoptosis in NSCLC (see Figure 6). However, the precise molecular mechanisms by which miR-34a mediates these activities need further elucidation in NSCLC. Furthermore, the possibility that other miRNAs may play an important role in the regulation of cell cycle progression in NSCLC is not discounted. It becomes clear that miR-34a has a fundamental role in senescence and apoptosis by targeting Myc and BCL-2 in NSCLC [6].

## 5. Conclusions

In summary, our model agrees with the experimental results associated with the individual influence of ANRIL and/or UFC1 Vs miR-34a in cell fate decisions in NSCLC cells. We highlighted a very complex scenario between the lncRNA-miRNA-mRNA. Furthermore, we predicted the three new biological positive circuits involving miR-34a. Worthy of note is the prediction of a new role for miR-34a in preventing NSCLC progression through the induction of apoptosis and senescence in NSCLC at the G1/S checkpoint. Therefore, our results support the idea that ANRIL and/or UFC1 is an attractive target of drug development in tumor growth and the aggressive proliferation of NSCLC, and that a reasonable outcome can be achieved through the miRNA-34a/Myc pathway.

## Figures and Tables

**Table 1:** Agreement between proposed Boolean Network and experimental data from the literature. Ectopic expression (E1) represents gain of function (GoF) and Knockdown (KO) represents loss of function (LoF) of the corresponding molecule. Cases for which no experimental data were found are indicated by ‘?’.

Stimulus/Perturbations	Model Response	Experimental Observations	Agreement	Cell Lines	References
In response to DNA damage, miR-34a vs. Myc in NSCLC	Negative correlation	Negative correlation	Yes	A549, H460	[6]
In response to DNA damage, miR-34a E1 in NSCLC	Inhibits the activity of Myc, HDAC1, and BCL2, and induces Senescence and Apoptosis	Represses Myc, and BCL2 inducing Senescence and Apoptosis	Yes	A549, H460	[6]
In response to DNA damage, Myc KO in NSCLC	Induces the activity of miR-34a activating Senescence and Apoptosis	Overexpression of miR-34a induces Senescence and Apoptosis	Yes	A549, H460	[6]
In response to DNA damage, Myc E1 in NSCLC	Inhibits miR-34a expression and induces apoptosis	Represses miR-34a expression and induces apoptotic cell death	Yes	A549, H460	[6]
In response to DNA damage, ANRIL KO in NSCLC	Inactivation of ANRIL activating senescence and Apoptosis	Knockdown (KO) of ANRIL Inhibits proliferation and induced apoptosis and cell cycle arrest	Yes	A549, H1299	[2]
In response to DNA damage, UFC1 KO in NSCLC	Inactivation of UFC1 induces senescence and Apoptosis	Knockdown (KO) of UFC1 Inhibits proliferation and induced apoptosis and cell cycle arrest	Yes	A549, H1299	[3]
In response to DNA damage, ANRIL/UFC1 vs. miR-34a in NSCLC	Negative correlation	Negative correlation	-	A549, H460, H1299	?
In response to DNA damage, UFC1 KO in NSCLC	Inactivation of UFC1 triggers miR-34a activity induces Senescence and Apoptosis	Inhibits proliferation and induced apoptosis and senescence	-	A549, H460, H1299	?
In response to DNA damage, ANRIL KO in NSCLC	Inactivation of UFC1 stimulates miR-34a activity induces Senescence and Apoptosis	Inhibits proliferation and induced apoptosis and senescence	-	A549, H460, H1299	?

**Table 2:** Functional circuits of the model and experimental observations. Cases not studied experimentally are indicated by question marks (?).

Biological Circuits	References
**Positive**	
p53/miR-34a/HDAC1	[48]
E2F1/CDK2-CycE/RB	[49]
p21/Caspase3	[50]
Myc/p21	[51]
ATM/miR-34a/HDAC1	[12]
p53-A/p53-K	[35]
E2F1/Myc	[52]
miR-34a/E2F1/ANRIL	?
miR-34a/E2F1/UFC1	?
miR-34a/Myc/ANRIL	?
E2F1/ATM	[53]
**Negative**	
p53/Mdm2	[34]
E2F1/Sirt1	[54]
p53-A/p53-INP1	[35]
E2F1/ATM/miR-34a	[12]
p53/Wip1/ATM	[55]

**Table 3:** The evidence of the biochemical interactions defining the biological positive circuits is well-characterized in the literature.

Positive Circuits	Circuit Elements	Target	Direct/Indirect Interaction	References
miR-34a/E2F1/ANRIL	miR-34a	E2F1	Direct inhibition	[42]
	E2F1	ANRIL	Direct activation	[40]
	ANRIL	miR-34a	Direct inhibition	[10,56]
miR-34a/E2F1/UFC1	miR-34a	E2F1	Direct inhibition	[42]
	E2F1	UFC1	Direct activation	[11]
	UFC1	miR-34a	Direct inhibition	[47]
miR-34a/Myc/ANRIL	miR-34a	Myc	Direct inhibition	[6]
	Myc	ANRIL	Direct activation	[41]
	ANRIL	miR-34a	Direct inhibition	[10,56]

**Table 4:** Perturbations of the three newly identified positive circuits. Ectopic expression (E1) represents gain-of-function (GoF), whereas, knockdown (KO) represents loss-of-function (LoF), respectively.

Positive Circuits	Perturbations	Phenotypes
miR-34a/E2F1/ANRIL	KO/KO/KO	Apoptosis
	E1/KO/KO	Senescence and Apoptosis
	KO/E1/KO	Apoptosis
	KO/KO/E1	Apoptosis
	E1/E1/KO	Apoptosis
	E1/KO/E1	Senescence and Apoptosis
	KO/E1/E1	Apoptosis
	E1/E1/E1	Apoptosis
miR-34a/E2F1/UFC1	KO/KO/KO	Apoptosis
	E1/KO/KO	Senescence and Apoptosis
	KO/E1/KO	Apoptosis
	KO/KO/E1	Apoptosis
	E1/E1/KO	Apoptosis
	E1/KO/E1	Senescence and Apoptosis
	KO/E1/E1	Apoptosis
	E1/E1/E1	Apoptosis
miR-34a/Myc/ANRIL	KO/KO/KO	Apoptosis
	E1/KO/KO	Senescence and Apoptosis
	KO/E1/KO	Apoptosis
	KO/KO/E1	Apoptosis
	E1/E1/KO	Apoptosis
	E1/KO/E1	Senescence and Apoptosis
	KO/E1/E1	Apoptosis
	E1/E1/E1	Apoptosis

## Data Availability

Data is contained within the article or Supplementary Material.

## Supplementary Materials

The following supporting information can be downloaded at: https://www.mdpi.com/article/10.3390/biom12030420/s1, Data File S1: The GINsim 3.0.0b code of the Boolean Network.; File S1: Predictions of the Boolean Network.; Table S1: Official name of the molecules and the proposed logical rules controlling the nodes of the Network.; Table S2: Functional circuits of the Boolean Network.
