# Supplementary material for: Dynamical Analysis of a Boolean Network Model of the Oncogene Role of lncRNA ANRIL and lncRNA UFC1 in Non-Small Cell Lung Cancer
Source: Biomolecules. 2022 Mar 9;12(3):420. doi: 10.3390/biom12030420 (PMC8946683; doi:10.3390/biom12030420)
Supplement: Supplementary file 1 [file biomolecules-12-00420-s001.zip › Data_File_S1.pdf]

Dynamical analysis of a Boolean Network model of the oncogene role of lncRNA-ANRIL and lncRNA-UFC1 in non-small cell lung cancer

Data File S1

Shantanu Gupta[1, +, *] and Ronaldo F. Hashimoto[1, +]

[1]Instituto de Matemática e Estatística, Departamento de Ciência da Computação, Universidade de

São Paulo, Rua do Matão 1010, 05508-090, São Paulo - SP, Brasil

[*]Corresponding author (e-mail: shantanu.gupta@ime.usp.br)

[+]These authors contributed equally to this work

Code of the model in .ginml file format used for the GINsim 3.0.0b software.

```xml
<?xml version="1.0" encoding="UTF-8"?>
<!DOCTYPE gxl SYSTEM "http://gin.univ-mrs.fr/GINsim/GINML_2_1.dtd">
<gxl>
  <graph id="defaultID" class="regulatory" nodeorder="DNA_Damage ATM p38MAPK Mdm2 p53 Wip1 p53_A p53_K miR_34a p21 p53_INP1 Myc Cdc25A cdk46_CycD cdk2_CycE RB E2F1 ANRIL UFC1 EZH2 KLF2 PTEN AKT Sirt_1 HDAC1 PUMA BCL2 BAX Caspase3 Proliferation Senescence Apoptosis">
    <node id="DNA_Damage" maxvalue="1">
      <parameter val="1" idActiveInteractions="DNA_Damage:DNA_Damage:1"/>
      <annotation>
        <comment>DNA_Damage is an input node marked with an auto-regulation.</comment>
      </annotation>
    </node>
    <node id="ATM" maxvalue="1">
      <parameter val="1" idActiveInteractions="DNA_Damage:ATM:1"/>
      <parameter val="1" idActiveInteractions="DNA_Damage:ATM:1 HDAC1:ATM:1"/>
      <parameter val="1" idActiveInteractions="DNA_Damage:ATM:1 E2F1:ATM:1"/>
      <parameter val="1" idActiveInteractions="DNA_Damage:ATM:1 E2F1:ATM:1 HDAC1:ATM:1"/>
      <parameter val="1" idActiveInteractions="DNA_Damage:ATM:1 Wip1:ATM:1"/>
      <parameter val="1" idActiveInteractions="DNA_Damage:ATM:1 Wip1:ATM:1 E2F1:ATM:1"/>
      <parameter val="1" idActiveInteractions="DNA_Damage:ATM:1 Wip1:ATM:1 E2F1:ATM:1 HDAC1:ATM:1"/>
    </node>
    <node id="p38MAPK" maxvalue="1">
      <parameter val="1" idActiveInteractions="ATM:p38MAPK:1"/>
    </node>
    <node id="Mdm2" maxvalue="1">
      <parameter val="1"/>
      <parameter val="1" idActiveInteractions="p53:Mdm2:1"/>
      <parameter val="1" idActiveInteractions="p53:Mdm2:1 Wip1:Mdm2:1"/>
    </node>
    <node id="p53" maxvalue="1">
      <parameter val="1" idActiveInteractions="p38MAPK:p53:1"/>
      <parameter val="1" idActiveInteractions="ATM:p53:1"/>
      <parameter val="1" idActiveInteractions="ATM:p53:1 HDAC1:p53:1"/>
      <parameter val="1" idActiveInteractions="ATM:p53:1 Mdm2:p53:1"/>
      <parameter val="1" idActiveInteractions="ATM:p53:1 Mdm2:p53:1 HDAC1:p53:1"/>
      <parameter val="1" idActiveInteractions="ATM:p53:1 p38MAPK:p53:1"/>
      <parameter val="1" idActiveInteractions="ATM:p53:1 p38MAPK:p53:1 HDAC1:p53:1"/>
      <parameter val="1" idActiveInteractions="ATM:p53:1 p38MAPK:p53:1 Mdm2:p53:1"/>
      <parameter val="1" idActiveInteractions="ATM:p53:1 p38MAPK:p53:1 Mdm2:p53:1 HDAC1:p53:1"/>
    </node>
    <node id="Wip1" maxvalue="1">
      <parameter val="1" idActiveInteractions="p53_A:Wip1:1"/>
    </node>
    <node id="p53_A" maxvalue="1">
      <parameter val="1"/>
      <parameter val="1" idActiveInteractions="p53:p53_A:1"/>
      <parameter val="1" idActiveInteractions="p53:p53_A:1 p53_INP1:p53_A:1"/>
    </node>
    <node id="p53_K" maxvalue="1">
      <parameter val="1" idActiveInteractions="p53:p53_K:1"/>
      <parameter val="1" idActiveInteractions="p53:p53_K:1 Sirt_1:p53_K:1"/>
      <parameter val="1" idActiveInteractions="p53:p53_K:1 Wip1:p53_K:1"/>
    </node>
    <node id="miR_34a" maxvalue="1">
      <parameter val="1" idActiveInteractions="p53:miR_34a:1"/>
```

```xml
      <parameter val="1" idActiveInteractions="ATM:miR_34a:1"/>
      <parameter val="1" idActiveInteractions="ATM:miR_34a:1 p53:miR_34a:1"/>
    </node>
    <node id="p21" maxvalue="1">
      <parameter val="1" idActiveInteractions="p53_A:p21:1"/>
      <parameter val="1" idActiveInteractions="p53_A:p21:1 Caspase3:p21:1"/>
      <parameter val="1" idActiveInteractions="p53_A:p21:1 HDAC1:p21:1"/>
      <parameter val="1" idActiveInteractions="p53_A:p21:1 HDAC1:p21:1 Caspase3:p21:1"/>
      <parameter val="1" idActiveInteractions="p53_A:p21:1 AKT:p21:1"/>
      <parameter val="1" idActiveInteractions="p53_A:p21:1 AKT:p21:1 Caspase3:p21:1"/>
      <parameter val="1" idActiveInteractions="p53_A:p21:1 AKT:p21:1 HDAC1:p21:1"/>
      <parameter val="1" idActiveInteractions="p53_A:p21:1 AKT:p21:1 HDAC1:p21:1 Caspase3:p21:1"/>
      <parameter val="1" idActiveInteractions="p53_A:p21:1 KLF2:p21:1"/>
      <parameter val="1" idActiveInteractions="p53_A:p21:1 KLF2:p21:1 Caspase3:p21:1"/>
      <parameter val="1" idActiveInteractions="p53_A:p21:1 KLF2:p21:1 HDAC1:p21:1"/>
      <parameter val="1" idActiveInteractions="p53_A:p21:1 KLF2:p21:1 HDAC1:p21:1 Caspase3:p21:1"/>
      <parameter val="1" idActiveInteractions="p53_A:p21:1 KLF2:p21:1 AKT:p21:1"/>
      <parameter val="1" idActiveInteractions="p53_A:p21:1 KLF2:p21:1 AKT:p21:1 Caspase3:p21:1"/>
      <parameter val="1" idActiveInteractions="p53_A:p21:1 KLF2:p21:1 AKT:p21:1 HDAC1:p21:1"/>
      <parameter val="1" idActiveInteractions="p53_A:p21:1 KLF2:p21:1 AKT:p21:1 HDAC1:p21:1 Caspase3:p21:1"/>
      <parameter val="1" idActiveInteractions="p53_A:p21:1 EZH2:p21:1"/>
      <parameter val="1" idActiveInteractions="p53_A:p21:1 EZH2:p21:1 Caspase3:p21:1"/>
      <parameter val="1" idActiveInteractions="p53_A:p21:1 EZH2:p21:1 HDAC1:p21:1"/>
      <parameter val="1" idActiveInteractions="p53_A:p21:1 EZH2:p21:1 HDAC1:p21:1 Caspase3:p21:1"/>
      <parameter val="1" idActiveInteractions="p53_A:p21:1 EZH2:p21:1 AKT:p21:1"/>
      <parameter val="1" idActiveInteractions="p53_A:p21:1 EZH2:p21:1 AKT:p21:1 Caspase3:p21:1"/>
      <parameter val="1" idActiveInteractions="p53_A:p21:1 EZH2:p21:1 AKT:p21:1 HDAC1:p21:1"/>
      <parameter val="1" idActiveInteractions="p53_A:p21:1 EZH2:p21:1 AKT:p21:1 HDAC1:p21:1 Caspase3:p21:1"/>
      <parameter val="1" idActiveInteractions="p53_A:p21:1 EZH2:p21:1 KLF2:p21:1"/>
      <parameter val="1" idActiveInteractions="p53_A:p21:1 EZH2:p21:1 KLF2:p21:1 Caspase3:p21:1"/>
      <parameter val="1" idActiveInteractions="p53_A:p21:1 EZH2:p21:1 KLF2:p21:1 HDAC1:p21:1"/>
      <parameter val="1" idActiveInteractions="p53_A:p21:1 EZH2:p21:1 KLF2:p21:1 HDAC1:p21:1 Caspase3:p21:1"/>
      <parameter val="1" idActiveInteractions="p53_A:p21:1 EZH2:p21:1 KLF2:p21:1 AKT:p21:1"/>
      <parameter val="1" idActiveInteractions="p53_A:p21:1 EZH2:p21:1 KLF2:p21:1 AKT:p21:1 Caspase3:p21:1"/>
      <parameter val="1" idActiveInteractions="p53_A:p21:1 EZH2:p21:1 KLF2:p21:1 AKT:p21:1 HDAC1:p21:1"/>
      <parameter val="1" idActiveInteractions="p53_A:p21:1 EZH2:p21:1 KLF2:p21:1 AKT:p21:1 HDAC1:p21:1 Caspase3:p21:1"/>
      <parameter val="1" idActiveInteractions="p53_A:p21:1 Myc:p21:1"/>
      <parameter val="1" idActiveInteractions="p53_A:p21:1 Myc:p21:1 Caspase3:p21:1"/>
      <parameter val="1" idActiveInteractions="p53_A:p21:1 Myc:p21:1 HDAC1:p21:1"/>
      <parameter val="1" idActiveInteractions="p53_A:p21:1 Myc:p21:1 HDAC1:p21:1 Caspase3:p21:1"/>
      <parameter val="1" idActiveInteractions="p53_A:p21:1 Myc:p21:1 AKT:p21:1"/>
      <parameter val="1" idActiveInteractions="p53_A:p21:1 Myc:p21:1 AKT:p21:1 Caspase3:p21:1"/>
      <parameter val="1" idActiveInteractions="p53_A:p21:1 Myc:p21:1 AKT:p21:1 HDAC1:p21:1"/>
      <parameter val="1" idActiveInteractions="p53_A:p21:1 Myc:p21:1 AKT:p21:1 HDAC1:p21:1 Caspase3:p21:1"/>
      <parameter val="1" idActiveInteractions="p53_A:p21:1 Myc:p21:1 KLF2:p21:1"/>
      <parameter val="1" idActiveInteractions="p53_A:p21:1 Myc:p21:1 KLF2:p21:1 Caspase3:p21:1
```

```
"/>
    <parameter val="1" idActiveInteractions="p53_A:p21:1 Myc:p21:1 KLF2:p21:1 HDAC1:p21:1"/>
    <parameter val="1" idActiveInteractions="p53_A:p21:1 Myc:p21:1 KLF2:p21:1 HDAC1:p21:1 Caspase3:p21:1"/>
    <parameter val="1" idActiveInteractions="p53_A:p21:1 Myc:p21:1 KLF2:p21:1 AKT:p21:1"/>
    <parameter val="1" idActiveInteractions="p53_A:p21:1 Myc:p21:1 KLF2:p21:1 AKT:p21:1 Caspase3:p21:1"/>
    <parameter val="1" idActiveInteractions="p53_A:p21:1 Myc:p21:1 KLF2:p21:1 AKT:p21:1 HDAC1:p21:1"/>
    <parameter val="1" idActiveInteractions="p53_A:p21:1 Myc:p21:1 KLF2:p21:1 AKT:p21:1 HDAC1:p21:1 Caspase3:p21:1"/>
    <parameter val="1" idActiveInteractions="p53_A:p21:1 Myc:p21:1 EZH2:p21:1"/>
    <parameter val="1" idActiveInteractions="p53_A:p21:1 Myc:p21:1 EZH2:p21:1 Caspase3:p21:1"/>
    <parameter val="1" idActiveInteractions="p53_A:p21:1 Myc:p21:1 EZH2:p21:1 HDAC1:p21:1"/>
    <parameter val="1" idActiveInteractions="p53_A:p21:1 Myc:p21:1 EZH2:p21:1 HDAC1:p21:1 Caspase3:p21:1"/>
    <parameter val="1" idActiveInteractions="p53_A:p21:1 Myc:p21:1 EZH2:p21:1 AKT:p21:1"/>
    <parameter val="1" idActiveInteractions="p53_A:p21:1 Myc:p21:1 EZH2:p21:1 AKT:p21:1 Caspase3:p21:1"/>
    <parameter val="1" idActiveInteractions="p53_A:p21:1 Myc:p21:1 EZH2:p21:1 AKT:p21:1 HDAC1:p21:1"/>
    <parameter val="1" idActiveInteractions="p53_A:p21:1 Myc:p21:1 EZH2:p21:1 AKT:p21:1 HDAC1:p21:1 Caspase3:p21:1"/>
    <parameter val="1" idActiveInteractions="p53_A:p21:1 Myc:p21:1 EZH2:p21:1 KLF2:p21:1"/>
    <parameter val="1" idActiveInteractions="p53_A:p21:1 Myc:p21:1 EZH2:p21:1 KLF2:p21:1 Caspase3:p21:1"/>
    <parameter val="1" idActiveInteractions="p53_A:p21:1 Myc:p21:1 EZH2:p21:1 KLF2:p21:1 HDAC1:p21:1"/>
    <parameter val="1" idActiveInteractions="p53_A:p21:1 Myc:p21:1 EZH2:p21:1 KLF2:p21:1 HDAC1:p21:1 Caspase3:p21:1"/>
    <parameter val="1" idActiveInteractions="p53_A:p21:1 Myc:p21:1 EZH2:p21:1 KLF2:p21:1 AKT:p21:1"/>
    <parameter val="1" idActiveInteractions="p53_A:p21:1 Myc:p21:1 EZH2:p21:1 KLF2:p21:1 AKT:p21:1 Caspase3:p21:1"/>
    <parameter val="1" idActiveInteractions="p53_A:p21:1 Myc:p21:1 EZH2:p21:1 KLF2:p21:1 AKT:p21:1 HDAC1:p21:1"/>
    <parameter val="1" idActiveInteractions="p53_A:p21:1 Myc:p21:1 EZH2:p21:1 KLF2:p21:1 AKT:p21:1 HDAC1:p21:1 Caspase3:p21:1"/>
    <parameter val="1" idActiveInteractions="p38MAPK:p21:1 KLF2:p21:1"/>
    <parameter val="1" idActiveInteractions="p38MAPK:p21:1 p53_A:p21:1"/>
    <parameter val="1" idActiveInteractions="p38MAPK:p21:1 p53_A:p21:1 Caspase3:p21:1"/>
    <parameter val="1" idActiveInteractions="p38MAPK:p21:1 p53_A:p21:1 HDAC1:p21:1"/>
    <parameter val="1" idActiveInteractions="p38MAPK:p21:1 p53_A:p21:1 HDAC1:p21:1 Caspase3:p21:1"/>
    <parameter val="1" idActiveInteractions="p38MAPK:p21:1 p53_A:p21:1 AKT:p21:1"/>
    <parameter val="1" idActiveInteractions="p38MAPK:p21:1 p53_A:p21:1 AKT:p21:1 Caspase3:p21:1"/>
    <parameter val="1" idActiveInteractions="p38MAPK:p21:1 p53_A:p21:1 AKT:p21:1 HDAC1:p21:1"/>
    <parameter val="1" idActiveInteractions="p38MAPK:p21:1 p53_A:p21:1 AKT:p21:1 HDAC1:p21:1 Caspase3:p21:1"/>
    <parameter val="1" idActiveInteractions="p38MAPK:p21:1 p53_A:p21:1 KLF2:p21:1"/>
    <parameter val="1" idActiveInteractions="p38MAPK:p21:1 p53_A:p21:1 KLF2:p21:1 Caspase3:p21:1"/>
    <parameter val="1" idActiveInteractions="p38MAPK:p21:1 p53_A:p21:1 KLF2:p21:1 HDAC1:p21:1"/>
    <parameter val="1" idActiveInteractions="p38MAPK:p21:1 p53_A:p21:1 KLF2:p21:1 HDAC1:p21:1 Caspase3:p21:1"/>
    <parameter val="1" idActiveInteractions="p38MAPK:p21:1 p53_A:p21:1 KLF2:p21:1 AKT:p21:1"/>
    <parameter val="1" idActiveInteractions="p38MAPK:p21:1 p53_A:p21:1 KLF2:p21:1 AKT:p21:1
```

```
Caspase3:p21:1"/>
<parameter val="1" idActiveInteractions="p38MAPK:p21:1 p53_A:p21:1 KLF2:p21:1 AKT:p21:1 HDAC1:p21:1"/>
<parameter val="1" idActiveInteractions="p38MAPK:p21:1 p53_A:p21:1 KLF2:p21:1 AKT:p21:1 HDAC1:p21:1 Caspase3:p21:1"/>
<parameter val="1" idActiveInteractions="p38MAPK:p21:1 p53_A:p21:1 EZH2:p21:1"/>
<parameter val="1" idActiveInteractions="p38MAPK:p21:1 p53_A:p21:1 EZH2:p21:1 Caspase3:p21:1"/>
<parameter val="1" idActiveInteractions="p38MAPK:p21:1 p53_A:p21:1 EZH2:p21:1 HDAC1:p21:1"/>
<parameter val="1" idActiveInteractions="p38MAPK:p21:1 p53_A:p21:1 EZH2:p21:1 HDAC1:p21:1 Caspase3:p21:1"/>
<parameter val="1" idActiveInteractions="p38MAPK:p21:1 p53_A:p21:1 EZH2:p21:1 AKT:p21:1"/>
<parameter val="1" idActiveInteractions="p38MAPK:p21:1 p53_A:p21:1 EZH2:p21:1 AKT:p21:1 Caspase3:p21:1"/>
<parameter val="1" idActiveInteractions="p38MAPK:p21:1 p53_A:p21:1 EZH2:p21:1 AKT:p21:1 HDAC1:p21:1"/>
<parameter val="1" idActiveInteractions="p38MAPK:p21:1 p53_A:p21:1 EZH2:p21:1 AKT:p21:1 HDAC1:p21:1 Caspase3:p21:1"/>
<parameter val="1" idActiveInteractions="p38MAPK:p21:1 p53_A:p21:1 EZH2:p21:1 KLF2:p21:1"/>
<parameter val="1" idActiveInteractions="p38MAPK:p21:1 p53_A:p21:1 EZH2:p21:1 KLF2:p21:1 Caspase3:p21:1"/>
<parameter val="1" idActiveInteractions="p38MAPK:p21:1 p53_A:p21:1 EZH2:p21:1 KLF2:p21:1 HDAC1:p21:1"/>
<parameter val="1" idActiveInteractions="p38MAPK:p21:1 p53_A:p21:1 EZH2:p21:1 KLF2:p21:1 HDAC1:p21:1 Caspase3:p21:1"/>
<parameter val="1" idActiveInteractions="p38MAPK:p21:1 p53_A:p21:1 EZH2:p21:1 KLF2:p21:1 AKT:p21:1"/>
<parameter val="1" idActiveInteractions="p38MAPK:p21:1 p53_A:p21:1 EZH2:p21:1 KLF2:p21:1 AKT:p21:1 Caspase3:p21:1"/>
<parameter val="1" idActiveInteractions="p38MAPK:p21:1 p53_A:p21:1 EZH2:p21:1 KLF2:p21:1 AKT:p21:1 HDAC1:p21:1"/>
<parameter val="1" idActiveInteractions="p38MAPK:p21:1 p53_A:p21:1 EZH2:p21:1 KLF2:p21:1 AKT:p21:1 HDAC1:p21:1 Caspase3:p21:1"/>
<parameter val="1" idActiveInteractions="p38MAPK:p21:1 p53_A:p21:1 Myc:p21:1"/>
<parameter val="1" idActiveInteractions="p38MAPK:p21:1 p53_A:p21:1 Myc:p21:1 Caspase3:p21:1"/>
<parameter val="1" idActiveInteractions="p38MAPK:p21:1 p53_A:p21:1 Myc:p21:1 HDAC1:p21:1"/>
<parameter val="1" idActiveInteractions="p38MAPK:p21:1 p53_A:p21:1 Myc:p21:1 HDAC1:p21:1 Caspase3:p21:1"/>
<parameter val="1" idActiveInteractions="p38MAPK:p21:1 p53_A:p21:1 Myc:p21:1 AKT:p21:1"/>
<parameter val="1" idActiveInteractions="p38MAPK:p21:1 p53_A:p21:1 Myc:p21:1 AKT:p21:1 Caspase3:p21:1"/>
<parameter val="1" idActiveInteractions="p38MAPK:p21:1 p53_A:p21:1 Myc:p21:1 AKT:p21:1 HDAC1:p21:1"/>
<parameter val="1" idActiveInteractions="p38MAPK:p21:1 p53_A:p21:1 Myc:p21:1 AKT:p21:1 HDAC1:p21:1 Caspase3:p21:1"/>
<parameter val="1" idActiveInteractions="p38MAPK:p21:1 p53_A:p21:1 Myc:p21:1 KLF2:p21:1"/>
<parameter val="1" idActiveInteractions="p38MAPK:p21:1 p53_A:p21:1 Myc:p21:1 KLF2:p21:1 Caspase3:p21:1"/>
<parameter val="1" idActiveInteractions="p38MAPK:p21:1 p53_A:p21:1 Myc:p21:1 KLF2:p21:1 HDAC1:p21:1"/>
<parameter val="1" idActiveInteractions="p38MAPK:p21:1 p53_A:p21:1 Myc:p21:1 KLF2:p21:1 HDAC1:p21:1 Caspase3:p21:1"/>
<parameter val="1" idActiveInteractions="p38MAPK:p21:1 p53_A:p21:1 Myc:p21:1 KLF2:p21:1 AKT:p21:1"/>
<parameter val="1" idActiveInteractions="p38MAPK:p21:1 p53_A:p21:1 Myc:p21:1 KLF2:p21:1
```

```
            AKT:p21:1 Caspase3:p21:1"/>
        <parameter val="1" idActiveInteractions="p38MAPK:p21:1 p53_A:p21:1 Myc:p21:1 KLF2:p21:1
            AKT:p21:1 HDAC1:p21:1"/>
        <parameter val="1" idActiveInteractions="p38MAPK:p21:1 p53_A:p21:1 Myc:p21:1 KLF2:p21:1
            AKT:p21:1 HDAC1:p21:1 Caspase3:p21:1"/>
        <parameter val="1" idActiveInteractions="p38MAPK:p21:1 p53_A:p21:1 Myc:p21:1 EZH2:p21:1"
            />
        <parameter val="1" idActiveInteractions="p38MAPK:p21:1 p53_A:p21:1 Myc:p21:1 EZH2:p21:1
            Caspase3:p21:1"/>
        <parameter val="1" idActiveInteractions="p38MAPK:p21:1 p53_A:p21:1 Myc:p21:1 EZH2:p21:1
            HDAC1:p21:1"/>
        <parameter val="1" idActiveInteractions="p38MAPK:p21:1 p53_A:p21:1 Myc:p21:1 EZH2:p21:1
            HDAC1:p21:1 Caspase3:p21:1"/>
        <parameter val="1" idActiveInteractions="p38MAPK:p21:1 p53_A:p21:1 Myc:p21:1 EZH2:p21:1
            AKT:p21:1"/>
        <parameter val="1" idActiveInteractions="p38MAPK:p21:1 p53_A:p21:1 Myc:p21:1 EZH2:p21:1
            AKT:p21:1 Caspase3:p21:1"/>
        <parameter val="1" idActiveInteractions="p38MAPK:p21:1 p53_A:p21:1 Myc:p21:1 EZH2:p21:1
            AKT:p21:1 HDAC1:p21:1"/>
        <parameter val="1" idActiveInteractions="p38MAPK:p21:1 p53_A:p21:1 Myc:p21:1 EZH2:p21:1
            AKT:p21:1 HDAC1:p21:1 Caspase3:p21:1"/>
        <parameter val="1" idActiveInteractions="p38MAPK:p21:1 p53_A:p21:1 Myc:p21:1 EZH2:p21:1
            KLF2:p21:1"/>
        <parameter val="1" idActiveInteractions="p38MAPK:p21:1 p53_A:p21:1 Myc:p21:1 EZH2:p21:1
            KLF2:p21:1 Caspase3:p21:1"/>
        <parameter val="1" idActiveInteractions="p38MAPK:p21:1 p53_A:p21:1 Myc:p21:1 EZH2:p21:1
            KLF2:p21:1 HDAC1:p21:1"/>
        <parameter val="1" idActiveInteractions="p38MAPK:p21:1 p53_A:p21:1 Myc:p21:1 EZH2:p21:1
            KLF2:p21:1 HDAC1:p21:1 Caspase3:p21:1"/>
        <parameter val="1" idActiveInteractions="p38MAPK:p21:1 p53_A:p21:1 Myc:p21:1 EZH2:p21:1
            KLF2:p21:1 AKT:p21:1"/>
        <parameter val="1" idActiveInteractions="p38MAPK:p21:1 p53_A:p21:1 Myc:p21:1 EZH2:p21:1
            KLF2:p21:1 AKT:p21:1 Caspase3:p21:1"/>
        <parameter val="1" idActiveInteractions="p38MAPK:p21:1 p53_A:p21:1 Myc:p21:1 EZH2:p21:1
            KLF2:p21:1 AKT:p21:1 HDAC1:p21:1"/>
        <parameter val="1" idActiveInteractions="p38MAPK:p21:1 p53_A:p21:1 Myc:p21:1 EZH2:p21:1
            KLF2:p21:1 AKT:p21:1 HDAC1:p21:1 Caspase3:p21:1"/>
    </node>
    <node id="p53_INP1" maxvalue="1">
        <parameter val="1" idActiveInteractions="p53_K:p53_INP1:1"/>
        <parameter val="1" idActiveInteractions="p53_A:p53_INP1:1"/>
        <parameter val="1" idActiveInteractions="p53_A:p53_INP1:1 p53_K:p53_INP1:1"/>
    </node>
    <node id="Myc" maxvalue="1">
        <parameter val="1"/>
        <parameter val="1" idActiveInteractions="E2F1:Myc:1"/>
        <parameter val="1" idActiveInteractions="miR_34a:Myc:1 E2F1:Myc:1"/>
        <parameter val="1" idActiveInteractions="p38MAPK:Myc:1"/>
        <parameter val="1" idActiveInteractions="p38MAPK:Myc:1 E2F1:Myc:1"/>
        <parameter val="1" idActiveInteractions="p38MAPK:Myc:1 miR_34a:Myc:1"/>
        <parameter val="1" idActiveInteractions="p38MAPK:Myc:1 miR_34a:Myc:1 E2F1:Myc:1"/>
    </node>
    <node id="Cdc25A" maxvalue="1">
        <parameter val="1"/>
        <parameter val="1" idActiveInteractions="miR_34a:Cdc25A:1"/>
        <parameter val="1" idActiveInteractions="p38MAPK:Cdc25A:1"/>
        <parameter val="1" idActiveInteractions="ATM:Cdc25A:1"/>
        <parameter val="1" idActiveInteractions="ATM:Cdc25A:1 miR_34a:Cdc25A:1"/>
    </node>
    <node id="cdk46_CycD" maxvalue="1">
        <parameter val="1" idActiveInteractions="Cdc25A:cdk46_CycD:1"/>
    </node>
```

```
<node id="cdk2_CycE" maxvalue="1">
  <parameter val="1" idActiveInteractions="Cdc25A:cdk2_CycE:1 E2F1:cdk2_CycE:1"/>
</node>
<node id="RB" maxvalue="1">
  <parameter val="1"/>
</node>
<node id="E2F1" maxvalue="1">
  <parameter val="1"/>
  <parameter val="1" idActiveInteractions="Sirt_1:E2F1:1"/>
  <parameter val="1" idActiveInteractions="Cdc25A:E2F1:1"/>
  <parameter val="1" idActiveInteractions="Cdc25A:E2F1:1 Sirt_1:E2F1:1"/>
  <parameter val="1" idActiveInteractions="Myc:E2F1:1"/>
  <parameter val="1" idActiveInteractions="Myc:E2F1:1 Sirt_1:E2F1:1"/>
  <parameter val="1" idActiveInteractions="Myc:E2F1:1 RB:E2F1:1"/>
  <parameter val="1" idActiveInteractions="Myc:E2F1:1 RB:E2F1:1 Sirt_1:E2F1:1"/>
  <parameter val="1" idActiveInteractions="Myc:E2F1:1 Cdc25A:E2F1:1"/>
  <parameter val="1" idActiveInteractions="Myc:E2F1:1 Cdc25A:E2F1:1 Sirt_1:E2F1:1"/>
  <parameter val="1" idActiveInteractions="Myc:E2F1:1 Cdc25A:E2F1:1 RB:E2F1:1"/>
  <parameter val="1" idActiveInteractions="Myc:E2F1:1 Cdc25A:E2F1:1 RB:E2F1:1 Sirt_1:E2F1:1"/>
  <parameter val="1" idActiveInteractions="miR_34a:E2F1:1"/>
  <parameter val="1" idActiveInteractions="miR_34a:E2F1:1 Cdc25A:E2F1:1"/>
  <parameter val="1" idActiveInteractions="miR_34a:E2F1:1 Myc:E2F1:1"/>
  <parameter val="1" idActiveInteractions="miR_34a:E2F1:1 Myc:E2F1:1 Sirt_1:E2F1:1"/>
  <parameter val="1" idActiveInteractions="miR_34a:E2F1:1 Myc:E2F1:1 RB:E2F1:1"/>
  <parameter val="1" idActiveInteractions="miR_34a:E2F1:1 Myc:E2F1:1 RB:E2F1:1 Sirt_1:E2F1:1"/>
  <parameter val="1" idActiveInteractions="miR_34a:E2F1:1 Myc:E2F1:1 Cdc25A:E2F1:1"/>
  <parameter val="1" idActiveInteractions="miR_34a:E2F1:1 Myc:E2F1:1 Cdc25A:E2F1:1 Sirt_1:E2F1:1"/>
  <parameter val="1" idActiveInteractions="miR_34a:E2F1:1 Myc:E2F1:1 Cdc25A:E2F1:1 RB:E2F1:1"/>
  <parameter val="1" idActiveInteractions="miR_34a:E2F1:1 Myc:E2F1:1 Cdc25A:E2F1:1 RB:E2F1:1 Sirt_1:E2F1:1"/>
  <parameter val="1" idActiveInteractions="ATM:E2F1:1"/>
  <parameter val="1" idActiveInteractions="ATM:E2F1:1 Sirt_1:E2F1:1"/>
  <parameter val="1" idActiveInteractions="ATM:E2F1:1 Cdc25A:E2F1:1"/>
  <parameter val="1" idActiveInteractions="ATM:E2F1:1 Cdc25A:E2F1:1 Sirt_1:E2F1:1"/>
  <parameter val="1" idActiveInteractions="ATM:E2F1:1 Myc:E2F1:1"/>
  <parameter val="1" idActiveInteractions="ATM:E2F1:1 Myc:E2F1:1 Sirt_1:E2F1:1"/>
  <parameter val="1" idActiveInteractions="ATM:E2F1:1 Myc:E2F1:1 RB:E2F1:1"/>
  <parameter val="1" idActiveInteractions="ATM:E2F1:1 Myc:E2F1:1 RB:E2F1:1 Sirt_1:E2F1:1"/>
  <parameter val="1" idActiveInteractions="ATM:E2F1:1 Myc:E2F1:1 Cdc25A:E2F1:1"/>
  <parameter val="1" idActiveInteractions="ATM:E2F1:1 Myc:E2F1:1 Cdc25A:E2F1:1 Sirt_1:E2F1:1"/>
  <parameter val="1" idActiveInteractions="ATM:E2F1:1 Myc:E2F1:1 Cdc25A:E2F1:1 RB:E2F1:1"/>
  <parameter val="1" idActiveInteractions="ATM:E2F1:1 Myc:E2F1:1 Cdc25A:E2F1:1 RB:E2F1:1 Sirt_1:E2F1:1"/>
  <parameter val="1" idActiveInteractions="ATM:E2F1:1 miR_34a:E2F1:1"/>
  <parameter val="1" idActiveInteractions="ATM:E2F1:1 miR_34a:E2F1:1 Cdc25A:E2F1:1"/>
  <parameter val="1" idActiveInteractions="ATM:E2F1:1 miR_34a:E2F1:1 Cdc25A:E2F1:1 Sirt_1:E2F1:1"/>
  <parameter val="1" idActiveInteractions="ATM:E2F1:1 miR_34a:E2F1:1 Myc:E2F1:1"/>
  <parameter val="1" idActiveInteractions="ATM:E2F1:1 miR_34a:E2F1:1 Myc:E2F1:1 Sirt_1:E2F1:1"/>
  <parameter val="1" idActiveInteractions="ATM:E2F1:1 miR_34a:E2F1:1 Myc:E2F1:1 RB:E2F1:1"/>
  <parameter val="1" idActiveInteractions="ATM:E2F1:1 miR_34a:E2F1:1 Myc:E2F1:1 RB:E2F1:1 Sirt_1:E2F1:1"/>
  <parameter val="1" idActiveInteractions="ATM:E2F1:1 miR_34a:E2F1:1 Myc:E2F1:1
```

```
        Cdc25A:E2F1:1"/>
    <parameter val="1" idActiveInteractions="ATM:E2F1:1 miR_34a:E2F1:1 Myc:E2F1:1
        Cdc25A:E2F1:1 Sirt_1:E2F1:1"/>
    <parameter val="1" idActiveInteractions="ATM:E2F1:1 miR_34a:E2F1:1 Myc:E2F1:1
        Cdc25A:E2F1:1 RB:E2F1:1"/>
    <parameter val="1" idActiveInteractions="ATM:E2F1:1 miR_34a:E2F1:1 Myc:E2F1:1
        Cdc25A:E2F1:1 RB:E2F1:1 Sirt_1:E2F1:1"/>
  </node>
  <node id="ANRIL" maxvalue="1">
    <parameter val="1" idActiveInteractions="E2F1:ANRIL:1"/>
    <parameter val="1" idActiveInteractions="Myc:ANRIL:1"/>
    <parameter val="1" idActiveInteractions="Myc:ANRIL:1 E2F1:ANRIL:1"/>
  </node>
  <node id="UFC1" maxvalue="1">
    <parameter val="1" idActiveInteractions="E2F1:UFC1:1"/>
  </node>
  <node id="EZH2" maxvalue="1">
    <parameter val="1" idActiveInteractions="Myc:EZH2:1 ANRIL:EZH2:1 UFC1:EZH2:1"/>
  </node>
  <node id="KLF2" maxvalue="1">
    <parameter val="1"/>
  </node>
  <node id="PTEN" maxvalue="1">
    <parameter val="1" idActiveInteractions="p53_K:PTEN:1"/>
  </node>
  <node id="AKT" maxvalue="1">
    <parameter val="1" idActiveInteractions="BCL2:AKT:1"/>
  </node>
  <node id="Sirt_1" maxvalue="1">
    <parameter val="1" idActiveInteractions="E2F1:Sirt_1:1"/>
    <parameter val="1" idActiveInteractions="E2F1:Sirt_1:1 HDAC1:Sirt_1:1"/>
    <parameter val="1" idActiveInteractions="miR_34a:Sirt_1:1 E2F1:Sirt_1:1"/>
  </node>
  <node id="HDAC1" maxvalue="1">
    <parameter val="1"/>
    <parameter val="1" idActiveInteractions="Sirt_1:HDAC1:1"/>
    <parameter val="1" idActiveInteractions="miR_34a:HDAC1:1"/>
    <parameter val="1" idActiveInteractions="miR_34a:HDAC1:1 Sirt_1:HDAC1:1"/>
    <parameter val="1" idActiveInteractions="DNA_Damage:HDAC1:1"/>
  </node>
  <node id="PUMA" maxvalue="1">
    <parameter val="1" idActiveInteractions="p53_K:PUMA:1"/>
  </node>
  <node id="BCL2" maxvalue="1">
    <parameter val="1"/>
  </node>
  <node id="BAX" maxvalue="1">
    <parameter val="1" idActiveInteractions="p53_K:BAX:1"/>
  </node>
  <node id="Caspase3" maxvalue="1">
    <parameter val="1" idActiveInteractions="BAX:Caspase3:1"/>
    <parameter val="1" idActiveInteractions="BCL2:Caspase3:1 BAX:Caspase3:1"/>
    <parameter val="1" idActiveInteractions="p21:Caspase3:1 BAX:Caspase3:1"/>
  </node>
  <node id="Proliferation" maxvalue="1">
    <parameter val="1" idActiveInteractions="E2F1:Proliferation:1"/>
  </node>
  <node id="Senescence" maxvalue="1">
    <parameter val="1" idActiveInteractions="p21:Senescence:1"/>
  </node>
  <node id="Apoptosis" maxvalue="1">
    <parameter val="1" idActiveInteractions="Caspase3:Apoptosis:1"/>
```

```
</node>
<edge id="DNA_Damage:DNA_Damage:1" from="DNA_Damage" to="DNA_Damage" sign="positive" minvalue="1"/>
<edge id="DNA_Damage:ATM:1" from="DNA_Damage" to="ATM" sign="positive" minvalue="1"/>
<edge id="Wip1:ATM:1" from="Wip1" to="ATM" sign="negative" minvalue="1"/>
<edge id="E2F1:ATM:1" from="E2F1" to="ATM" sign="positive" minvalue="1"/>
<edge id="HDAC1:ATM:1" from="HDAC1" to="ATM" sign="negative" minvalue="1"/>
<edge id="ATM:p38MAPK:1" from="ATM" to="p38MAPK" sign="positive" minvalue="1"/>
<edge id="Wip1:p38MAPK:1" from="Wip1" to="p38MAPK" sign="negative" minvalue="1"/>
<edge id="ATM:Mdm2:1" from="ATM" to="Mdm2" sign="negative" minvalue="1"/>
<edge id="p53:Mdm2:1" from="p53" to="Mdm2" sign="positive" minvalue="1"/>
<edge id="Wip1:Mdm2:1" from="Wip1" to="Mdm2" sign="negative" minvalue="1"/>
<edge id="ATM:p53:1" from="ATM" to="p53" sign="positive" minvalue="1"/>
<edge id="p38MAPK:p53:1" from="p38MAPK" to="p53" sign="positive" minvalue="1"/>
<edge id="Mdm2:p53:1" from="Mdm2" to="p53" sign="negative" minvalue="1"/>
<edge id="HDAC1:p53:1" from="HDAC1" to="p53" sign="negative" minvalue="1"/>
<edge id="p53_A:Wip1:1" from="p53_A" to="Wip1" sign="positive" minvalue="1"/>
<edge id="p53:p53_A:1" from="p53" to="p53_A" sign="positive" minvalue="1"/>
<edge id="p53_K:p53_A:1" from="p53_K" to="p53_A" sign="negative" minvalue="1"/>
<edge id="p53_INP1:p53_A:1" from="p53_INP1" to="p53_A" sign="negative" minvalue="1"/>
<edge id="Sirt_1:p53_A:1" from="Sirt_1" to="p53_A" sign="negative" minvalue="1"/>
<edge id="p53:p53_K:1" from="p53" to="p53_K" sign="positive" minvalue="1"/>
<edge id="Wip1:p53_K:1" from="Wip1" to="p53_K" sign="negative" minvalue="1"/>
<edge id="p53_A:p53_K:1" from="p53_A" to="p53_K" sign="negative" minvalue="1"/>
<edge id="Sirt_1:p53_K:1" from="Sirt_1" to="p53_K" sign="negative" minvalue="1"/>
<edge id="ATM:miR_34a:1" from="ATM" to="miR_34a" sign="positive" minvalue="1"/>
<edge id="p53:miR_34a:1" from="p53" to="miR_34a" sign="positive" minvalue="1"/>
<edge id="ANRIL:miR_34a:1" from="ANRIL" to="miR_34a" sign="negative" minvalue="1"/>
<edge id="UFC1:miR_34a:1" from="UFC1" to="miR_34a" sign="negative" minvalue="1"/>
<edge id="EZH2:miR_34a:1" from="EZH2" to="miR_34a" sign="negative" minvalue="1"/>
<edge id="p38MAPK:p21:1" from="p38MAPK" to="p21" sign="positive" minvalue="1"/>
<edge id="p53_A:p21:1" from="p53_A" to="p21" sign="positive" minvalue="1"/>
<edge id="Myc:p21:1" from="Myc" to="p21" sign="negative" minvalue="1"/>
<edge id="EZH2:p21:1" from="EZH2" to="p21" sign="negative" minvalue="1"/>
<edge id="KLF2:p21:1" from="KLF2" to="p21" sign="positive" minvalue="1"/>
<edge id="AKT:p21:1" from="AKT" to="p21" sign="negative" minvalue="1"/>
<edge id="HDAC1:p21:1" from="HDAC1" to="p21" sign="negative" minvalue="1"/>
<edge id="Caspase3:p21:1" from="Caspase3" to="p21" sign="negative" minvalue="1"/>
<edge id="p53_A:p53_INP1:1" from="p53_A" to="p53_INP1" sign="positive" minvalue="1"/>
<edge id="p53_K:p53_INP1:1" from="p53_K" to="p53_INP1" sign="positive" minvalue="1"/>
<edge id="p38MAPK:Myc:1" from="p38MAPK" to="Myc" sign="positive" minvalue="1"/>
<edge id="miR_34a:Myc:1" from="miR_34a" to="Myc" sign="negative" minvalue="1"/>
<edge id="p21:Myc:1" from="p21" to="Myc" sign="negative" minvalue="1"/>
<edge id="RB:Myc:1" from="RB" to="Myc" sign="negative" minvalue="1"/>
<edge id="E2F1:Myc:1" from="E2F1" to="Myc" sign="positive" minvalue="1"/>
<edge id="ATM:Cdc25A:1" from="ATM" to="Cdc25A" sign="negative" minvalue="1"/>
<edge id="p38MAPK:Cdc25A:1" from="p38MAPK" to="Cdc25A" sign="negative" minvalue="1"/>
<edge id="miR_34a:Cdc25A:1" from="miR_34a" to="Cdc25A" sign="negative" minvalue="1"/>
<edge id="miR_34a:cdk46_CycD:1" from="miR_34a" to="cdk46_CycD" sign="negative" minvalue="1"/>
<edge id="p21:cdk46_CycD:1" from="p21" to="cdk46_CycD" sign="negative" minvalue="1"/>
<edge id="Cdc25A:cdk46_CycD:1" from="Cdc25A" to="cdk46_CycD" sign="positive" minvalue="1"/>
<edge id="miR_34a:cdk2_CycE:1" from="miR_34a" to="cdk2_CycE" sign="negative" minvalue="1"/>
<edge id="p21:cdk2_CycE:1" from="p21" to="cdk2_CycE" sign="negative" minvalue="1"/>
<edge id="Cdc25A:cdk2_CycE:1" from="Cdc25A" to="cdk2_CycE" sign="positive" minvalue="1"/>
<edge id="E2F1:cdk2_CycE:1" from="E2F1" to="cdk2_CycE" sign="positive" minvalue="1"/>
<edge id="cdk46_CycD:RB:1" from="cdk46_CycD" to="RB" sign="negative" minvalue="1"/>
<edge id="cdk2_CycE:RB:1" from="cdk2_CycE" to="RB" sign="negative" minvalue="1"/>
<edge id="ATM:E2F1:1" from="ATM" to="E2F1" sign="positive" minvalue="1"/>
<edge id="miR_34a:E2F1:1" from="miR_34a" to="E2F1" sign="negative" minvalue="1"/>
```

```
    <edge id="Myc:E2F1:1" from="Myc" to="E2F1" sign="positive" minvalue="1"/>
    <edge id="Cdc25A:E2F1:1" from="Cdc25A" to="E2F1" sign="positive" minvalue="1"/>
    <edge id="RB:E2F1:1" from="RB" to="E2F1" sign="negative" minvalue="1"/>
    <edge id="Sirt_1:E2F1:1" from="Sirt_1" to="E2F1" sign="negative" minvalue="1"/>
    <edge id="Myc:ANRIL:1" from="Myc" to="ANRIL" sign="positive" minvalue="1"/>
    <edge id="E2F1:ANRIL:1" from="E2F1" to="ANRIL" sign="positive" minvalue="1"/>
    <edge id="E2F1:UFC1:1" from="E2F1" to="UFC1" sign="positive" minvalue="1"/>
    <edge id="ATM:EZH2:1" from="ATM" to="EZH2" sign="negative" minvalue="1"/>
    <edge id="Myc:EZH2:1" from="Myc" to="EZH2" sign="positive" minvalue="1"/>
    <edge id="ANRIL:EZH2:1" from="ANRIL" to="EZH2" sign="positive" minvalue="1"/>
    <edge id="UFC1:EZH2:1" from="UFC1" to="EZH2" sign="positive" minvalue="1"/>
    <edge id="Myc:KLF2:1" from="Myc" to="KLF2" sign="negative" minvalue="1"/>
    <edge id="EZH2:KLF2:1" from="EZH2" to="KLF2" sign="negative" minvalue="1"/>
    <edge id="p53_K:PTEN:1" from="p53_K" to="PTEN" sign="positive" minvalue="1"/>
    <edge id="EZH2:PTEN:1" from="EZH2" to="PTEN" sign="negative" minvalue="1"/>
    <edge id="PTEN:AKT:1" from="PTEN" to="AKT" sign="negative" minvalue="1"/>
    <edge id="BCL2:AKT:1" from="BCL2" to="AKT" sign="positive" minvalue="1"/>
    <edge id="miR_34a:Sirt_1:1" from="miR_34a" to="Sirt_1" sign="negative" minvalue="1"/>
    <edge id="E2F1:Sirt_1:1" from="E2F1" to="Sirt_1" sign="positive" minvalue="1"/>
    <edge id="HDAC1:Sirt_1:1" from="HDAC1" to="Sirt_1" sign="negative" minvalue="1"/>
    <edge id="DNA_Damage:HDAC1:1" from="DNA_Damage" to="HDAC1" sign="negative" minvalue="1"/>
    <edge id="miR_34a:HDAC1:1" from="miR_34a" to="HDAC1" sign="negative" minvalue="1"/>
    <edge id="Sirt_1:HDAC1:1" from="Sirt_1" to="HDAC1" sign="negative" minvalue="1"/>
    <edge id="p53_K:PUMA:1" from="p53_K" to="PUMA" sign="positive" minvalue="1"/>
    <edge id="miR_34a:BCL2:1" from="miR_34a" to="BCL2" sign="negative" minvalue="1"/>
    <edge id="PUMA:BCL2:1" from="PUMA" to="BCL2" sign="negative" minvalue="1"/>
    <edge id="p53_K:BAX:1" from="p53_K" to="BAX" sign="positive" minvalue="1"/>
    <edge id="BCL2:BAX:1" from="BCL2" to="BAX" sign="negative" minvalue="1"/>
    <edge id="p21:Caspase3:1" from="p21" to="Caspase3" sign="negative" minvalue="1"/>
    <edge id="BCL2:Caspase3:1" from="BCL2" to="Caspase3" sign="negative" minvalue="1"/>
    <edge id="BAX:Caspase3:1" from="BAX" to="Caspase3" sign="positive" minvalue="1"/>
    <edge id="p53:Proliferation:1" from="p53" to="Proliferation" sign="negative" minvalue="1"/
        >
    <edge id="E2F1:Proliferation:1" from="E2F1" to="Proliferation" sign="positive" minvalue="1
        "/>
    <edge id="p21:Senescence:1" from="p21" to="Senescence" sign="positive" minvalue="1"/>
    <edge id="E2F1:Senescence:1" from="E2F1" to="Senescence" sign="negative" minvalue="1"/>
    <edge id="Caspase3:Apoptosis:1" from="Caspase3" to="Apoptosis" sign="positive" minvalue="1
        "/>
  </graph>
</gxl>
```
