# Supplementary material for: Dynamical Analysis of a Boolean Network Model of the Oncogene Role of lncRNA ANRIL and lncRNA UFC1 in Non-Small Cell Lung Cancer
Source: Biomolecules. 2022 Mar 9;12(3):420. doi: 10.3390/biom12030420 (PMC8946683; doi:10.3390/biom12030420)
Supplement: Supplementary file 1 [file biomolecules-12-00420-s001.zip › File_S1.pdf]

Dynamical analysis of a Boolean Network model of the oncogene role of lncRNA-ANRIL and lncRNA-UFC1 in non-small cell lung cancer

File S1

Shantanu Gupta[1, +, *] and Ronaldo F. Hashimoto[1, +]

[1]Instituto de Matemática e Estatística, Departamento de Ciência da Computação, Universidade de

São Paulo, Rua do Matão 1010, 05508-090, São Paulo - SP, Brasil

[*]Corresponding author (e-mail: shantanu.gupta@ime.usp.br)

[+]These authors contributed equally to this work

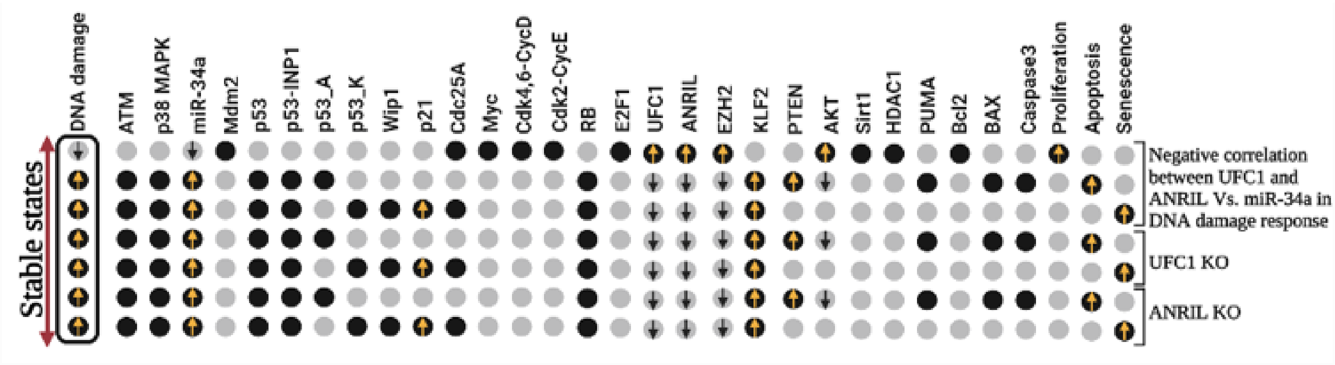

Figure S1. Prediction of the NSCLC model. Negative correlation between miR-34a, UFC1, and/or ANRIL in response to DNA damage. Gain-of-function (GoF) describes overexpression (E1) and loss-of-function (LoF) represents knockdown (KO) of the respective molecule. The leftmost column shows the DNA damage level (highlighted in the black box) and the rightmost column presents the model outputs: proliferation, senescence, and apoptosis. Each line represents a fixed point corresponding to the input. Gray cells indicate zero value, while black cells indicate activation (value 1). The first three stable states show a negative correlation between miR-34a and UFC1/ARIL in response to DNA damage. In more detail, first state: in the absence of DNA damage, miR-34a was downregulated, whereas Myc and UFC1/ANRIL were upregulated in inducing proliferation. Second and third stable states: in response to DNA damage, miR-34a was activated, which inhibits proliferation through induction of senescence and apoptosis. The next two states represent knockdown (KO) of UFC1: Forced knockdown of UFC1 induces miR-34a expression as well as inhibits proliferation through induction of senescence and apoptosis. The last two states represent knockdown (KO) of ANRIL: forced knockdown of ANRIL induces miR-34a expression as well as inhibits proliferation by activation of senescence and apoptosis. Orange-colored arrows indicate up-regulation of the molecule. Whereas, the inverted black arrow represents the downregulation of the molecule, respectively. This Figure was created by using BioRender.com.
